# CircGSK3B promotes RORA expression and suppresses gastric cancer progression through the prevention of EZH2 trans-inhibition

**DOI:** 10.1186/s13046-021-02136-w

**Published:** 2021-10-19

**Authors:** Xianxiong Ma, Hengyu Chen, Lei Li, Feng Yang, Chuanqing Wu, Kaixiong Tao

**Affiliations:** 1 Department of Gastrointestinal Surgery, Union Hospital, Tongji Medical College, Huazhong University of Science and Technology, Wuhan, 430022 China; 2 Department of Breast and Thyroid Surgery, The Second Affiliated Hospital of Hainan Medical University, Haikou, 570102 China; 3 Department of Breast and Thyroid Surgery, Union Hospital, Tongji Medical College, Huazhong University of Science and Technology, Wuhan, 430022 China; 4 Department of Orthopedic Surgery, The First Affiliated Hospital of Nanchang University, Nanchang, 330006 China

**Keywords:** CircGSK3B, EZH2, RORA, Gastric cancer, ArrayExpress, GEO

## Abstract

**Background:**

Circular RNAs (circRNAs) are a class of non-coding RNA that play critical roles in the development and pathogenesis of various cancers. The circRNA circGSK3B (hsa_circ_0003763) has been shown to enhance cell proliferation, migration, and invasion in hepatocellular carcinoma. However, the specific functions and underlying mechanistic involvement of circGSK3B in gastric cancer (GC) have not yet been explored. Our study aimed to investigate the effect of circGSK3B on the progression of GC and to identify any potential mechanisms underlying this process.

**Methods:**

CircRNA datasets associated with GC were obtained from the PubMed, GEO, and ArrayExpress databases, and circRNAs were validated via RT-qPCR and Sanger sequencing. Biotin-labeled RNA pull-down, mass spectrometry, RNA immunoprecipitation, and in vitro binding assays were employed to determine proteins demonstrating interactions with circGSK3B. Gene expression regulation was assessed through RT-qPCR, chromatin immunoprecipitation, and western blot assays. Gain- and loss-of-function assays were used to analyze any effects of circGSK3B and its partner regulatory molecule (EZH2) on the proliferation, invasion, and migration abilities of GC cells both in vitro and in vivo.

**Results:**

CircGSK3B was mainly identified in the nucleus. This circRNA was present at a reduced concentration in GC tissues and cell lines. Overexpression of circGSK3B was shown to inhibit the growth, invasion, and metastasis of GC cells both in vitro and in vivo. Mechanistically, circGSK3B directly interacted with EZH2, acting to suppress the binding of EZH2 and H3K27me3 to the *RORA* promoter, and leading to an elevation in RORA expression and ultimately the suppression of GC progression.

**Conclusions:**

CircGSK3B acts as a tumor suppressor, reducing EZH2 trans-inhibition and GC progression. This demonstrates the potential use of this RNA as a therapeutic target for GC.

**Supplementary Information:**

The online version contains supplementary material available at 10.1186/s13046-021-02136-w.

## Background

Gastric cancer (GC) is the fifth most frequently occurring cancer and is one of the major causes of cancer-associated death worldwide [1]. GC is the result of complex interactions among host genetic, environmental, and microbial factors [2]. Currently, surgical resection is a radical cure for GC patients, and the survival rate of advanced and metastatic GC remains poor [3]. Therefore, exploration of the molecular mechanisms underlying GC development and metastasis is urgently needed, as such studies might unveil new therapeutic targets.

Circular RNAs (circRNAs) are a class of regulatory RNA that form a covalently closed single-stranded loops, generated by precursor mRNA exon back-splicing [4–6]. Accumulating evidence has indicated that circRNAs play a critical regulatory role in the modulation of gene expression [7]. Recent studies have shown that circRNAs are involved in the development and pathogenesis of various cancers. For example, Yang et al. showed that hypoxia induces exosomal circRNA-promoted metastasis of colorectal cancer by targeting the GEF-H1/RhoA axis [8]. Furthermore, Xing et al. demonstrated that circIFI30 promotes the progression of triple-negative breast cancer, finding a correlation between this circRNA and prognosis [9]. Various studies have revealed that circRNAs are closely involved in the development of GC. For example, Xie et al. suggested that exosomal circSHKBP1 promotes GC progression through regulation of the miR-582-3p/HUR/VEGF axis and inhibition of HSP90 degradation [10]. Xin et al. demonstrated that circRNA_100782 promotes the proliferation and metastasis of GC cells by downregulating the tumor suppressor gene *Rb*, which occurs through the adsorption of miR-574-3p in a sponge form [11]. Peng et al. identified that circCUL2 regulates GC malignant transformation and cisplatin resistance via the modulation of autophagy activation mediated by miR-142-3p/ROCK2 [12]. A recent study reported that circRNA-circGSK3B (hsa_circ_0003763) promotes cell proliferation, migration, and invasion via miR-1265 sponging and the regulation of CAB39 expression in hepatocellular carcinoma [13]. Comparatively, the effects of circRNA_0003763 on GC progression and the mechanisms underlying this process have not yet been explored.

In our study, we searched circRNA expression profiles and discovered that circ_0003763 was downregulated in GC tissues and that this circRNA promoted GC progression. Mechanistically, circGSK3B directly interacted with EZH2, blocking the binding of EZH2 to the *RORA* promoter. This led to an elevation in RORA expression and the suppression of tumor progression, revealing the critical roles of circGSK3B and EZH2 in GC progression.

## Methods

### Patients and tissue specimens

Human tumor and adjacent non-tumor tissues (*n* = 56) were collected from GC patients who underwent gastrectomy at the Department of Gastrointestinal Surgery, Union Hospital of Huazhong University of Science and Technology (HUST) (Wuhan, China). All procedures were approved by the Ethics Committee of Union Hospital, HUST, and conducted according to the Declaration of Helsinki Principles. Prior written and informed consent was obtained from each patient.

### Integrated analysis of circRNA expression datasets

We performed a thorough search for available datasets in the electronic databases of PubMed, Gene Expression Omnibus (GEO) (Accession numbers: GSE78092, GSE83521, GSE89143, GSE93541, GSE100170, GSE121445, GSE122796, GSE141977, and GSE152309), and ArrayExpress (where no record was found) up to May 2021, using the following search terms: “(circRNA* OR circular) AND (gastric OR stomach)”. The workflow for proper microarray meta-analyses followed the recommendations of Ramasamy [14]. Only initial experimental studies that screened for different circRNAs from GC and adjacent/normal tissue samples in humans were included. Additional criteria for selection dictated that the included datasets should contain at least three samples of both GC and normal cells. Exclusion criteria included the following: (1) repeated records by the same institute; (2) non-microarray gene chips (for elimination of heterogeneity between throughput sequencing and microarray); (3) a non-whole-genome chip. After removing duplicated information, the combined datasets (GSE78092, GSE83521, GSE89143, GSE93541, GSE100170, and GSE141977, which contain six, 12, six, six, 10, and six samples, respectively) containing 23 GC and 23 normal GC tissue samples were generated (Additional file 1: Supplementary Table S1). The ‘limma’ R package was applied to preprocess the raw datasets [15] and all circRNA names were standardized according to circBase. Duplicate gene expression values were averaged.

The MetaDE package was used to identify differentially expressed circRNAs (DECs) between GC and normal tissues [16]. For integrated analysis, the mean and standard deviation (SD) filter thresholds were specified as 10%. Considering the different stringencies of the methods, Fisher’s method was performed for statistical analysis of significance; a modified t-test and permutation method were used to extrapolate the *P*-values [17]. One-sided tests were applied to classify the upregulated or downregulated DECs. *P* < 0.05 was considered statistically significant for these DECs.

### Cell culture

GC cell lines (SGC7901, BGC823, BGC803, AGS, and MKN45) and normal human gastric mucosal cells (GES-1) were purchased from the American Type Culture Collection (ATCC, https://www.atcc.org/). All cells were cultured in RPMI-1640 supplemented with 10% fetal bovine serum (FBS), 100 U/mL streptomycin, and 100 μg/mL penicillin (Invitrogen, Carlsbad, CA, USA) under standard conditions (5% CO_2_ at 37 °C).

### Gene overexpression and knockdown

Linear circGSK3B (286 bp) was synthesized by TSINGKE (Wuhan, China) and inserted into pLCDH-ciR (Geenseed Biotech Co., Guangzhou, China). Human EZH2 cDNA (2256 bp) was subcloned into CV186 (Genechem Co., Ltd., Shanghai, China), whereas its truncation variants were obtained by PCR amplification (Additional file 2: Supplementary Table S2) and inserted into pCMV-3Tag-1A or pGEX-6P-1 (Addgene, Cambridge, MA, USA), respectively. Oligonucleotides encoding short hairpin RNA (shRNA) against circRNAs and RORA (Additional file 2: Supplementary Table S3) were inserted into GV298 (GeneChem Co., Ltd). Lentiviral vectors were co-transfected with the packaging plasmids psPAX2 and pMD2G into HE K293T cells. Infectious lentiviruses were harvested at 36 and 60 h after transfection, followed with concentration by ultracentrifugation (2 h at 120,000×*g*). Stable cell lines were obtained by selection with puromycin. The EZH2 inhibitor (GSK126; MCE, HY-13470) was obtained from MedChemExpress company (New Jersey, USA).

### RNA extraction and quantitative real-time polymerase chain reaction (RT-qPCR)

Total RNA was extracted from the cells and tissues by applying the TRIzol reagent (Invitrogen, MA, USA) according to the manufacturer’s protocol. The isolated RNA was then reverse transcribed into cDNA with the PrimeScript RT enzyme Mix (TaKaRa Bio, Otsu, Japan). RT-qPCR was performed using SYBR Green PCR Master Mix (Vazyme) with an ABI Prism 7900 Sequence detection system (Applied Biosystems, Canada). *GAPDH* and U6 were used as internal controls, and each experiment was repeated in triplicate. Relative expression levels of circRNA and mRNA were normalized to those of the internal control and assessed employing the 2^−ΔΔCT^ method. The primers used in our study are listed in Additional file 2 (Supplementary Table S4).

### Western blot analysis

The total protein from cancer tissues and cells was isolated using radioimmunoprecipitation assay buffer, followed by concentration measurements using a bicinchoninic acid assay kit in accordance with the manufacturer’s instructions. Then, the protein samples were separated via sodium dodecyl sulfate-polyacrylamide gel electrophoresis and transferred to PVDF membranes (Millipore, USA). The membranes were blocked using 5% fat-free milk in TBST buffer for 1 h followed by incubation with primary antibodies (EZH2, #5246, CST; EED, #85322, CST; SUZ12, #3737, CST; H3K27me3, #9733, CST; β-catenin, ab32572, abcam; FLAG, ab1162, abcam; GST, sc-138, Santa Cruz; P-β-catenin, sc-57,535, Santa Cruz; RORA, sc-518,081, Santa Cruz; CCND1, ab134175, abcam; PCNA, ab29, abcam; Ki-67, ab16667, abcam; CD31, ab9498, abcam; GAPDH, ab8245, abcam) at 4 °C overnight. The next day, the membranes were washed three times with TBST and then incubated with a secondary antibody for 1 h. Finally, the proteins were visualized using the enhanced chemiluminescence system (Millipore). Each experiment was repeated in triplicate.

### Transwell assay

Transwell assays were employed to assess the migration and invasion capacity of GC cells. Briefly, the cells were placed in the upper chamber with serum-free medium while the bottom compartment was filled with culture medium containing 30% FBS. After incubation for 48 h, the GC cells that had invaded the lower chamber were fixed in 75% ethyl alcohol and then stained with 0.1% crystal violet. Finally, the invasive cells were counted manually via light microscopy.

### MTT assay

The proliferative capacity of GC cells was assessed using MTT assays in accordance with the manufacturer’s instructions. The target cells were added to a 96-well plate with 2 × 10^3^ cells per well and incubated for 1, 2, 3, 4, and 5 days at 37 °C. This was followed by the addition of 25 μL of MTT solution (5 mg/mL) to each well and incubation at 37 °C for 4 h. Then, 150 μL DMSO was used to dissolve the precipitates. Finally, the absorbance values were measured at 570 nm by utilizing a Multiskan™ GO microplate spectrophotometer (Thermo Fisher Scientific, Gillingham, UK).

### Flow cytometry

Flow cytometry was adopted to assess apoptotic cells and cell cycle distribution. Apoptosis assays (BD Pharmingen) were carried out in accordance with the manufacturer’s protocol. Briefly, after transfection for 48 h, the GC cells were digested and suspended in 1× binding buffer at a density of 1–5 × 10^6^ cells/mL. Subsequently, cells were harvested and dyed using annexin V-fluorescein isothiocyanate and propidium iodide in 6-well plates in the dark at 4 °C for 15 min. The samples were assessed using a BD flow cytometer (Franklin Lakes, NJ, USA). All experiments were performed in triplicate.

### RNA immunoprecipitation (RIP)

RIP assays were conducted with an RNA-binding protein immunoprecipitation kit (Millipore, Bedford, MA, USA) in accordance with the manufacturer’s instructions, with antibodies for EZH2 (1:100, #5246, CST), EED (1:100, #85322, CST), and SUZ12 (1:100, #3737, CST). Co-precipitated RNA was assessed by RT-qPCR assays using specific primers.

### Biotin-labeled RNA pull-down and mass spectrometry

RNA pull-down assays were conducted with the Pierce™ RNA 3′ end Desthiobiotinylation Kits (Thermo Scientific, USA) and Pierce™ magnetic RNA-protein pull-down kits (Thermo Scientific, USA) in accordance with the manufacturer’s instructions. The RNA-bound protein complex was eluted and evaluated through western blotting assays. Finally, mass spectrometry (MS) assays were carried out by SpecAlly Life Technology Co., Ltd. (Wuhan, China).

### RNA-fluorescence in situ hybridization (FISH)

RNA-FISH was performed using the Fluorescent In Situ Hybridization Kit (C10910, RiboBio) according to the manufacturer’s instructions. Biotin-labeled probes targeting circGSK3B (Additional file 2: Supplementary Table S3) were synthesized by RiboBio Technology Co. Ltd. Fluorescence excitation was recorded with a Zeiss confocal laser scanning microscope (LSM 880 with Airyscan, Carl Zeiss).

### RNA sequencing assay

Total RNA was extracted using TRIzol reagent (Invitrogen, MA, USA). Transcriptome sequencing on an Illumina Novaseq 6000 platform was carried out at Novogene Bioinformatics Technology Co., Ltd.

### Animal experimentation

In vivo experiments were conducted using BALB/c nude mice (male, 6-weeks-old). The mice were separated into three groups at random, with the investigator blinded to group allocation. To develop subcutaneous xenograft models, 2 × 10^6^ control, circGSK3B-downregulated, or circGSK3B-overexpressing MKN45 cells were resuspended in PBS and then subcutaneously injected into mice. One month later, mice were sacrificed and tumor weights were measured. All animal experiments were performed in compliance with the guidelines approved by the Institutional Animal Care Use Committee of Huazhong University of Science and Technology.

### Immunofluorescence

Immunofluorescence assays were conducted to assess the expression levels of circGSK3B and EZH2. GC cells were plated in six-well plates and incubated for 24 h after transfection. The cells were then washed three times using PBS and fixed with 4% paraformaldehyde at room temperature for 15 min. Then, these cells were permeabilized with 0.5% triton-X100-PBS for 15 min and incubated with primary antibodies (EZH2, 1:200, #5246, CST) overnight at 4 °C. The next day, the samples were treated with a secondary antibody for 1 h. Subsequently, cell nuclei were stained with 4′,6-diamidino-2-phenylindole (DAPI) for 10 min. Finally, cells were visualized with a fluorescence microscope.

### Chromatin Immunoprecipitation (ChIP) assay

ChIP analysis was performed with the Magna ChIP™ G Assay Kit in accordance with the manufacturer’s instructions. Briefly, the target GC cells were cross-linked using 37% formaldehyde for 10 min at room temperature; the chromatin was then sheared via sonication. The chromatin–protein complexes were immunoprecipitated using an anti-ChIP assay antibody (EZH2, 1:100, #5246, CST; H3K27me3, 1:50, #9733, CST) or an IgG control. The resulting DNA fragments were then employed as templates for RT-qPCR based on specific primers (Additional file 2: Supplementary Table S4).

### Statistical analysis

Data are presented as the mean ± SD, and statistical analyses were conducted with GraphPad Prism 5. The results were compared by a Student’s *t*-test or one-way analysis of variance. A value of *P* < 0.05 was considered statistically significant.

## Results

### CircGSK3B is downregulated in GC cells and tissues

**Fig. 1:**
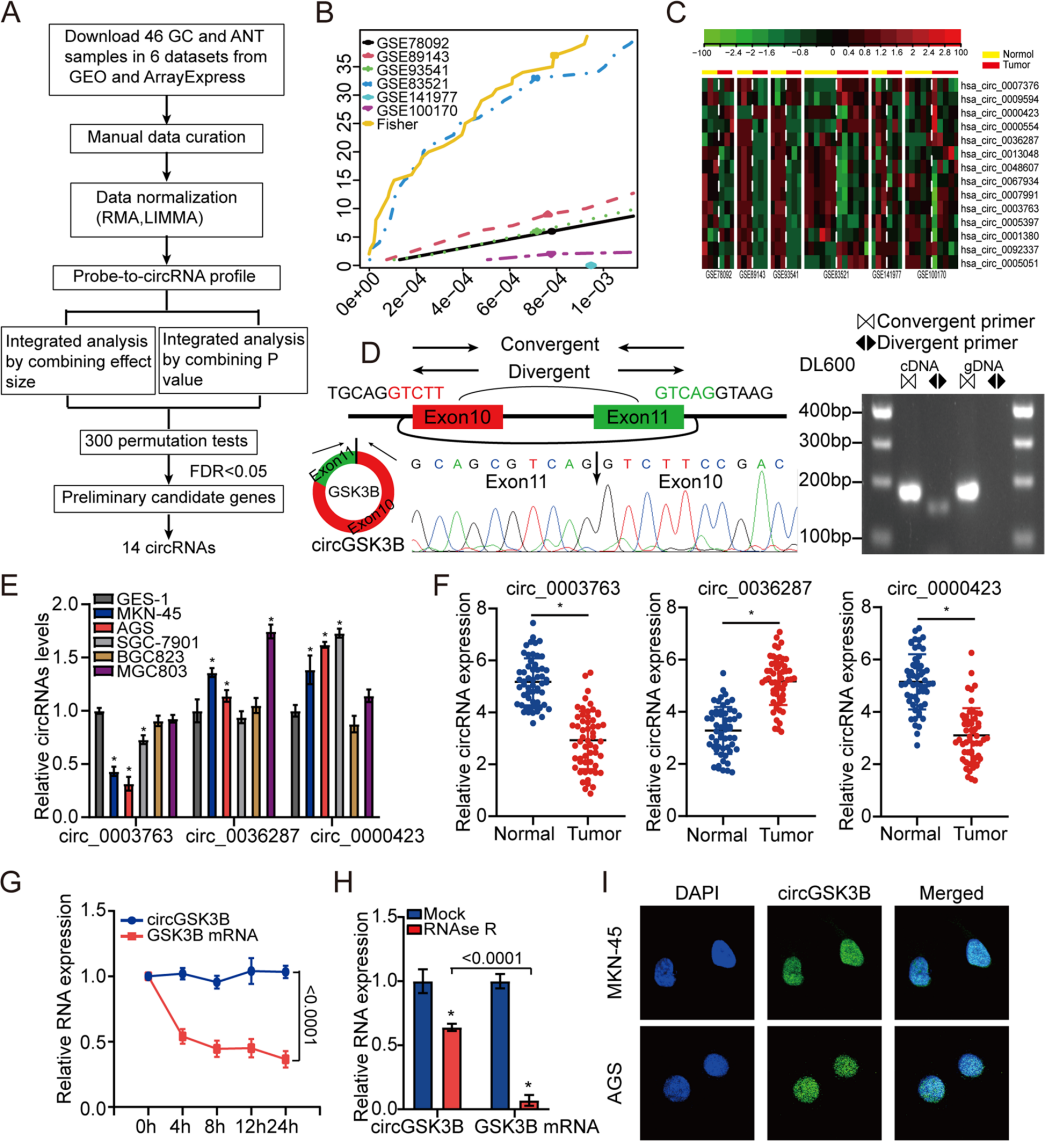
CircGSK3B is downregulated in GC cells and tissues. **a** Flow chart of circRNA selection. **b** The number of DECs against the *P*-value in the assessment of the 6 datasets. **c** Heatmap based on 14 DECs between GC and normal samples in the 6 datasets. **d** The genomic locus of circGSK3B. The expression level of circGSK3B was assessed via RT-PCR assay and Sanger sequencing. Arrows represent divergent primers targeting circGSK3B’s genome region (Left); RT-qPCR products using divergent primers indicating circularization of circGSK3B. cDNA represents complementary DNA. gDNA represents genomic DNA (Right). **e** A RT-qPCR assay was used to assess the expression of the three DECs in GES-1, SGC7901, MKN45, BGC823, AGS and MGC803 cells. **f** RT-qPCR analysis of circGSK3B in 56 GC and NC samples. **g** RT–qPCR analysis for the expression of circGSK3B and GSK3B mRNAs after treatment with Actinomycin D at the indicated time points in AGS cells. **h** RT–qPCR assays were used to assess the expression of circGSK3B and GSK3B mRNA in AGS cells after treatment using RNase R. Data represented as mean ± SD. Each experiment was performed five times; dot plot data points represent each independent experiment. The *P*-value was calculated using a two-tailed unpaired Student’s t-test. (**i**) The RNA-FISH assay revealed the nuclear localization of circGSK3B in MKN45 and AGS cells by an antisense probe (green). Nuclei were stained with DAPI

In total, 23 GC and 23 normal samples were obtained from six datasets via the GEO and ArrayExpress databases. The flow chart of the circRNA screening process is shown in Fig. 1a. The number of DECs and the associated combing results are presented in Fig. 1b, alongside the relevant *P*-value for each dataset [18]. After merging the expression data of the six datasets, 14 total DECs, between GC and normal samples, were determined and a heatmap was generated (Fig. 1c). Among these, only seven circRNAs were validated by PCR amplification using divergent primers from cDNA of GC cell lines (Additional file 3: Figure S1a). We performed Sanger sequencing using the PCR products of these seven circRNAs, and the results identified three circRNAs that were consistent with the sequence information in the NCBI database (Fig. 1d; Additional file 3: Figure S1b & c). The expression levels of these three circRNAs were assessed using RT-qPCR assays based on the five GC cell lines, the GES-1 cell line, GC tissue samples, and normal tissue samples. The results indicated that only circRNA_0003763 was consistently downregulated in GC tissues and GC cell lines. Therefore, this circRNA was selected for further analysis (Fig. 1e & f). CircRNA_0003763 was named circGSK3B, and the results showed that it was most significantly downregulated in MKN45 and AGS cell lines. Therefore, these two GC cell lines were selected for subsequent investigation. After treatment with actinomycin D, an inhibitor of transcription, RT-qPCR analysis showed that the half-life of circGSK3B exceeded 24 h, whereas that of the associated linear transcript was approximately 8 h (Fig. 1g). The resistance to digestion via RNase R exonuclease indicated that circGSK3B had a circular RNA structure (Fig. 1h). In addition, FISH assay analysis (Fig. 1i) suggested endogenous nuclear enrichment of circGSK3B in GC.

### CircGSK3B suppresses GC cell proliferation, invasion, and migration in vitro and in vivo

**Fig. 2:**
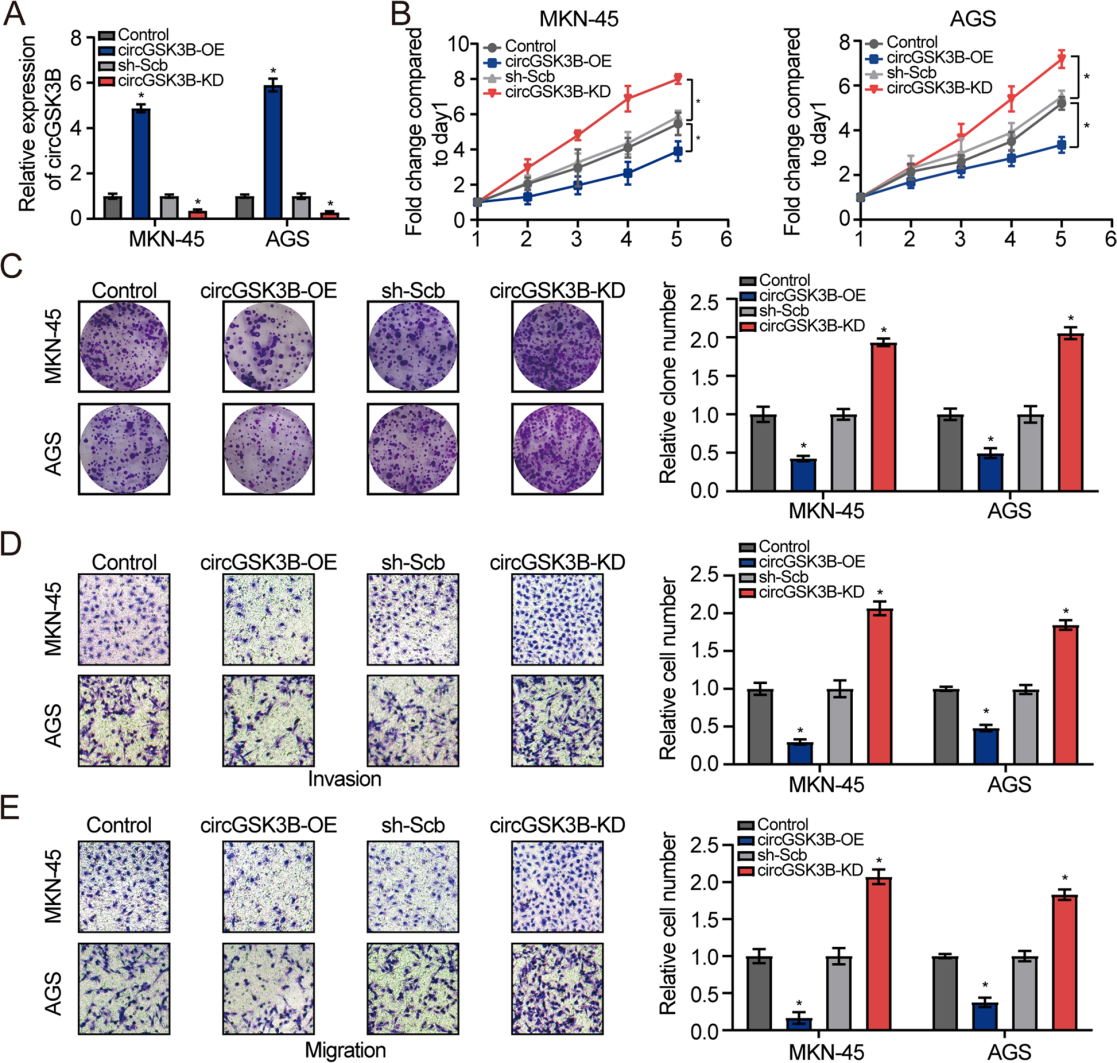
CircGSK3B suppresses GC cell proliferation, invasion and migration in vitro. **a** RT-qPCR assays for investigation of circGSK3B expression levels with circGSK3B vectors in MKN45 and AGS cells. **b, c** Cell proliferation was detected via cell viability and colony formation with variable circGSK3B expression in MKN45 and AGS cells. **d**, **e** Transwell assays were adopted to assess GC cell invasive and migratory capabilities with circGSK3B overexpression or knockdown in MKN45 and AGS cells

**Fig. 3:**
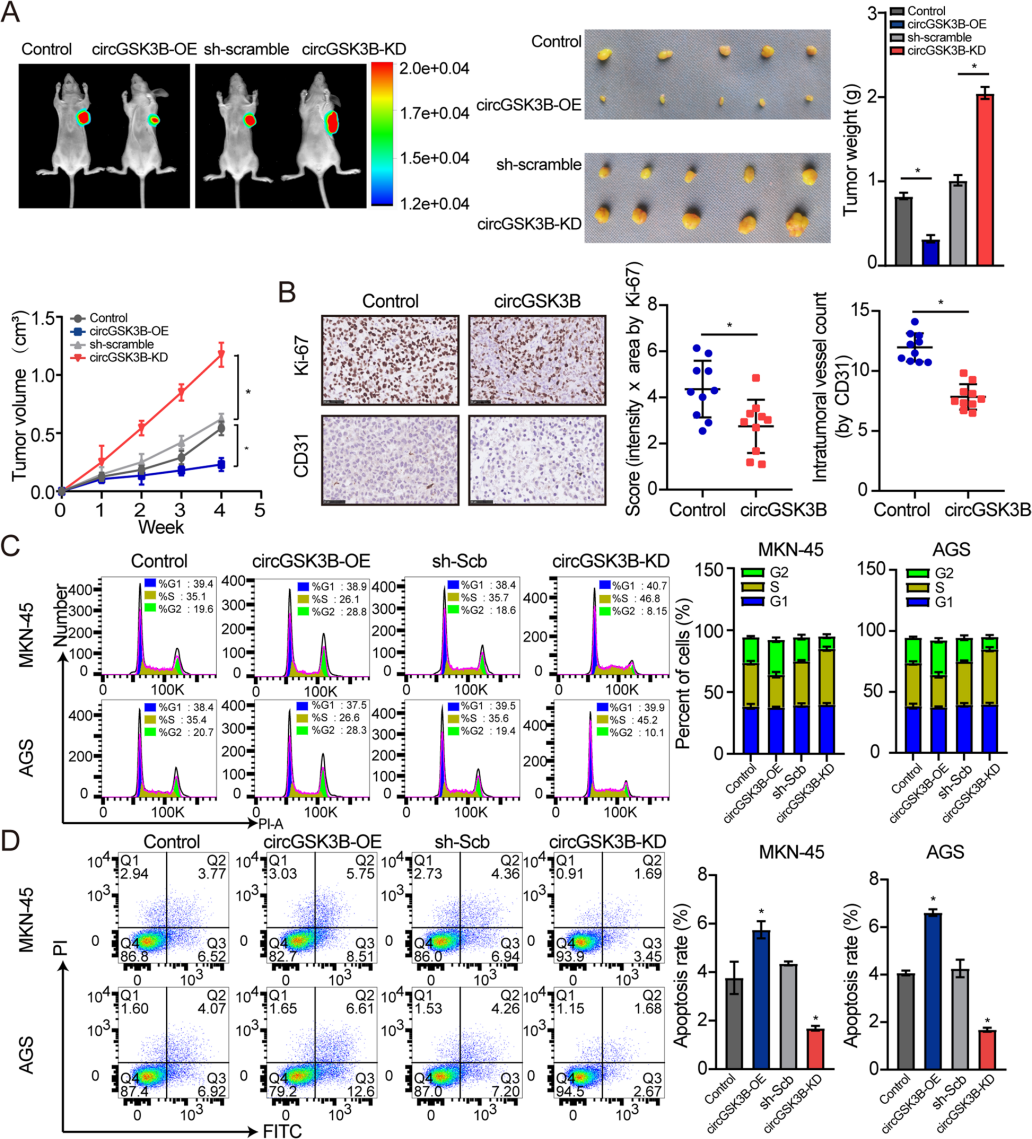
Effects of circGSK3B in vivo and on cell cycle and apoptosis. **a** Representative images (Top left and middle), in vivo growth curve (Bottom left), and weight at the end points (Top right) of xenografts formed by subcutaneous injection of MKN45 cells stably transfected with mock, circGSK3B, sh-Scb, or sh-circGSK3B #2 into the dorsal flanks of nude mice (*n* = 5 for each group). **b** Representative images (left panel) and quantification (right panel) of immunohistochemical staining showing the expression of Ki-67 and CD31 within xenograft tumors formed by hypodermic injection of MKN45 cells stably transfected with circ-Mock or circGSK3B (*n* = 5 for each group). **c** Flow cytometry was adopted to assess the impact of circGSK3B on the cell cycle of GC cells. **d** Flow cytometry was used to assess the effect of circGSK3B on apoptosis in GC cells

To assess the effect of circGSK3B on the two selected human GC cell lines, it was transfected into MKN45 and AGS cells. The results of RT-qPCR assays showed that circGSK3B was significantly overexpressed by the circGSK3B plasmid, and its expression was downregulated by the associated shRNA in MKN45 and AGS cells (Fig. 2a; Additional file 3: Figure S2a). Following circGSK3B knockdown, the proliferation, invasion, and migration abilities of GC cells were significantly increased. Comparatively, circGSK3B overexpression produced the reverse effect in MKN45 and AGS cells (Fig. 2b–e). Stable transfection with circGSK3B resulted in a significant decrease in the growth and weights of xenograft tumors formed by the subcutaneous injection of MKN45 cells into athymic nude mice (Fig. 3a). Furthermore, immunohistochemistry assays demonstrated that circGSK3B overexpression could cause a decrease in Ki-67 and CD31 expression (Fig. 3b), suggesting reduced cell proliferation. Taken together, these results show that circGSK3B can attenuate GC cell proliferation, migration, and invasion. However, high circGSK3B expression significantly suppressed GC cell cycle progression and enhanced apoptosis, whereas circGSK3B knockdown had the reverse effect (Fig. 3c & d).

### CircGSK3B interacts with EZH2 in GC cells

**Fig. 4:**
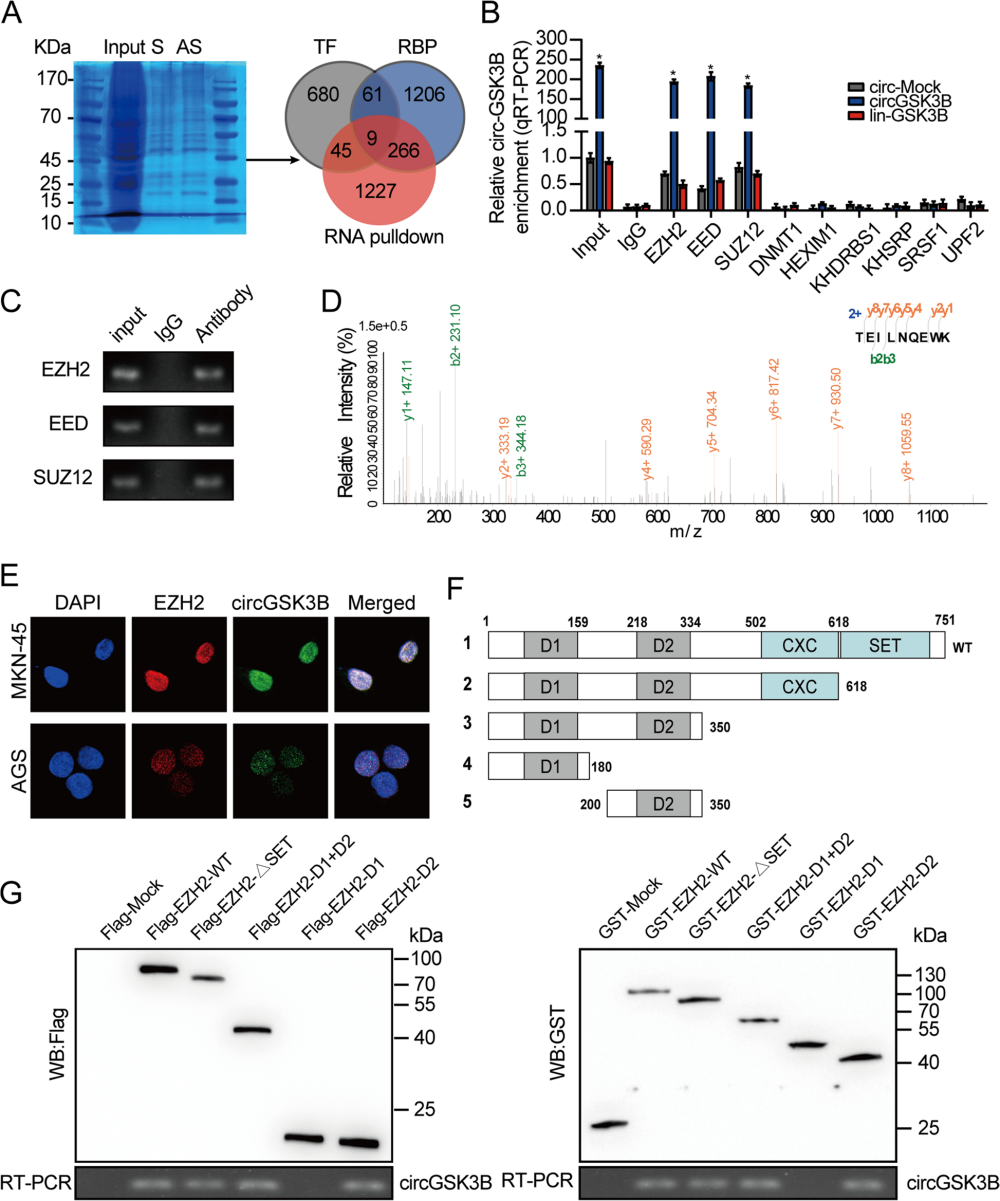
CircGSK3B interplayed with EZH2 protein in GC cells. **a** Coomassie bright blue staining (left panel), mass spectrometry analysis, as well as overlapping assays (Venn diagram, right panel) with developed RBP and TF databases suggesting proteins pulled down based on biotin-labeled antisense or sense forms of circGSK3B from the lysates of MKN45 cells. **b** RIP and real-time RT-qPCR assays were used to assess the relative interplay between circGSK3B and 9 proteins in MKN45 cells with circGSK3B or linear GSK3B, which were normalized to GC cells transfected using circ-Mock. **c** RIP assay with primer datasets revealing the interaction between circGSK3B and EZH2/EED/SUZ12 in MKN45 cells with circ-Mock, circGSK3B, or lin-GSK3B. **d** MS assay showing the EZH2 peptides pulled down by circGSK3B. **e** Dual RNA-FISH and immunofluorescence staining assays indicating the co-localization of circGSK3B (green) and EZH2 (red) in AGS and MKN-45 cells, with nuclei staining using DAPI (blue). **f** Schematic diagram indicating the domains of EZH2 truncations. **g** In vitro binding assay showing the enriched circGSK3B levels assessed based on RT-PCR (lower panel) after incubation using full-length or truncations versions of Flag-tagged or GST-tagged recombinant EZH2 protein validated by western blot (upper panel)

To explore the potential mechanism underlying the biological effects of circGSK3B, RIP assays and biotin-labeled RNA pull-down were employed to investigate the biological partners of circGSK3B. MS results indicated 1555 proteins with differential expression between the antisense and sense probe pull-down groups, which were used to investigate the intersection with developed RNA binding proteins (RBPs), as well as transcription factors (TFs). As a result, nine underlying circGSK3B-interacting molecules were identified (Fig. 4a; Additional file 2: Supplementary Table S5). RIP assays also indicated the binding of circGSK3B to EZH2, EED, and SUZ12, rather than DNMT1, HEMX1, KHDRBS1, KHSRP, SRSF1, or UPF2. (Fig. 4b; Additional file 3: Figure S2c & d). Moreover, transfection with circGSK3B, but not linear GSK3B, led to increased enrichment in RNA co-precipitated by EZH2, EED, or SUZ12 antibodies in MKN45 cells (Fig. 4c). The EZH2 peptides determined by MS assays are shown in Fig. 4d. The outcome of dual RNA-FISH and immunofluorescence assays verified the nuclear co-localization of circGSK3B and EZH2 in MKN45 and AGS cells (Fig. 4e), suggesting that they might play a role at the transcriptional level. Additionally, in vitro binding assays indicated that the D2 domain [200–350 amino acids] of the FLAG-tagged or GST-tagged EZH2 protein was crucial for its interaction with circGSK3B (Fig. 4f & g).

### CircGSK3B targets RORA expression through EZH2-mediated epigenetic regulation in GC cells

**Fig. 5:**
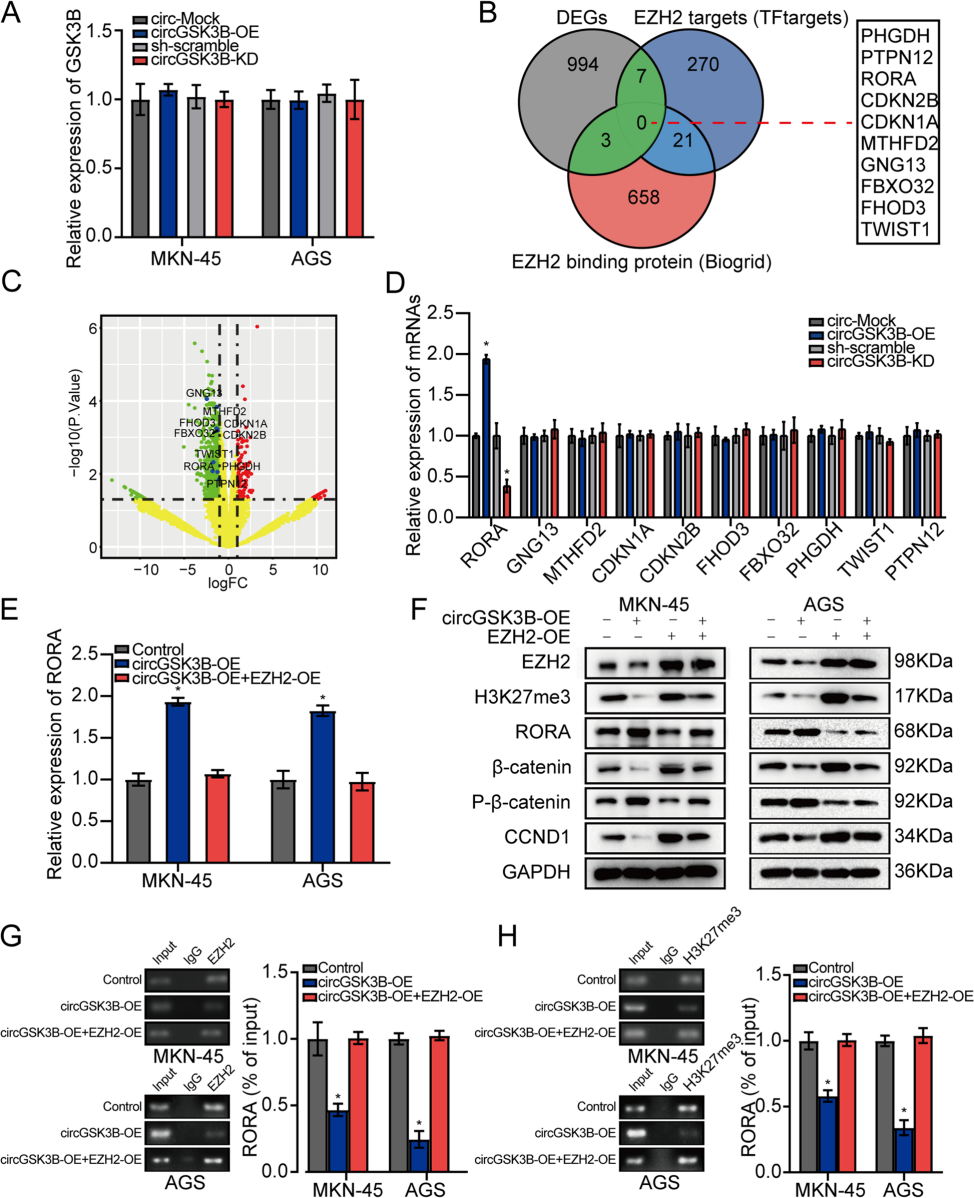
circGSK3B targeted RORA expression through EZH2-mediated epigenetic regulation in GC cells. **a** RT-qPCR analysis for GSK3B mRNA in AGS, MKN45 cells stably transfected with mock, circGSK3B, and sh-circGSK3B. **b** Overlapping analysis of DEGs between circGSK3B-OE and control group with EZH2 targets from the tftargets R package or EZH2 binding targets from Biogrid. **c** Volcano plot of the selected 10 DEGs in the RNAseq dataset. **d** RT-qPCR analysis for 10 screened mRNAs in MKN45 cells stably transfected with mock, circGSK3B, and sh-circGSK3B. **e** RT-qPCR analysis for RORA mRNA in AGS, MKN45 cells stably transfected with mock, circGSK3B, and circGSK3B + EZH2. **f** The effect of circGSK3B and EZH2 on *RORA*, H3K27me3, β-catenin, P-β-catenin, and *CCND1* (Canonical target gene of β-catenin) assessed by western blot assay. **g**, **h** ChIP assay showing the binding of EZH2 (**g**) and H3K27me3 (**h**) to the promoter of *RORA* assessed in MKN45 and AGS cells stably transfected with mock, circGSK3B, or circGSK3B + EZH2

To find the downstream target of circGSK3B, we first explored the potential cis-regulation of this circRNA, that is, whether circGSK3B could regulate its maternal gene *GSK3B*. RT-qPCR analysis was performed to assess the expression of *GSK3B* in AGS and MKN45 cells stably transfected with a mock circGSK3B (sh-circGSK3B). The result showed no significant change in *GSK3B* expression with circGSK3B overexpression or knockdown (Fig. 5a), indicating that circGSK3B did not have a cis-regulatory effect on the maternal *GSK3B* gene. We then used a high-throughput approach to find the targets of circGSK3B from the perspective of trans-regulation. This approach led to the identification of circGSK3B-induced differentially expressed genes (DEGs) by RNA-seq in MKN45 cells. We found 1004 DEGs (fold-change > 2, *P* < 0.05) upon circGSK3B overexpression, including 143 upregulated and 861 downregulated genes. Overlapping analysis of DEGs with EZH2-binding protein information from the BioGRID database [19] or predicted EZH2 targets from the tftargets package [20] identified 10 potential circGSK3B targets, specifically *GNG13*, *MTHFD2*, *FHOD3*, *CDKN1A*, *CDKN2B*, *FBXO32*, *TWIST1*, *RORA*, *PHGDH*, and *PTPN12* (Fig. 5b & c; Additional file 2: Supplementary Table S6). RT-qPCR analysis was performed to assess the expression level of these genes, and the results indicated that overexpression or knockdown of circGSK3B increased or reduced the expression of *RORA*, respectively, in MKN45 cells, whereas the other nine genes were unaffected (Fig. 5d). Notably, circGSK3B overexpression significantly increased the expression of *RORA*, which was reversed by EZH2 overexpression (Fig. 5e; Additional file 3: Figure S3a). Previous studies have shown that EZH2 functions as a gene silencer, mainly through the trimethylation of histone H3 lysine 27 (H3K27me3) [21] and that RORA attenuates β-catenin signaling through PKC-alpha-dependent phosphorylation [22]. To understand the molecular mechanisms regulating RORA expression, the effects of circGSK3B and EZH2 on *RORA*, H3K27me3, β-catenin, P-β-catenin, and cyclin D1 (*CCND1*, the canonical target gene of β-catenin) were analyzed by western blot assays. As indicated in Fig. 5f, EZH2 overexpression could significantly increase the expression level of H3K27me3, β-catenin, and *CCND1* and decrease the expression level of *RORA* and P-β-catenin, which could be reversed by circGSK3B overexpression. Similarly, the effects of EZH2 knockdown on RORA and β-catenin mediated by GSK-126 could be rescued by circGSK3B knockdown (Additional file 3: Figure S3b). Consistently, ChIP-qPCR analysis showed that in both MKN45 and AGS cells with circGSK3B and EZH2 overexpression, the binding of EZH2 or H3K27me3 to the promoter of *RORA* was markedly reduced (*P* < 0.05; Fig. 5g & h; Additional file 3: Figure S3c & d). This occurred despite the overall binding between *RORA* and EZH2 or H3K27me3 being relatively low, as indicated by the percentage of input. These data suggest that circGSK3B could compete with histones to bind EZH2, participating in the EZH2-mediated epigenetic repression of *RORA* in malignant GC cells.

### Effect of circGSK3B, EZH2, and RORA on growth and aggressiveness of GC cells

**Fig. 6:**
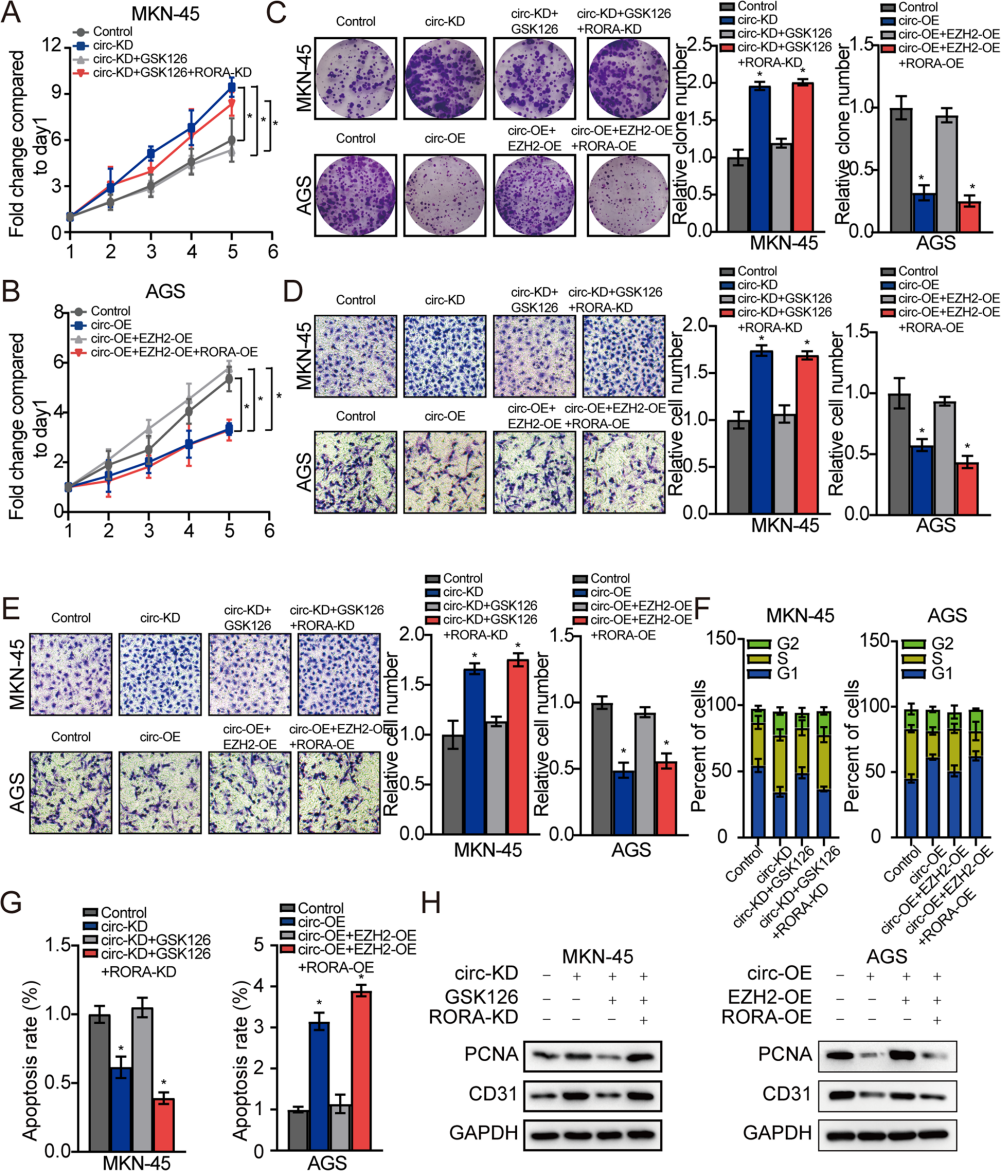
The effect of circGSK3B, EZH2 and RORA on growth and aggressiveness of GC cells. **a**, **b**, **c** The effect of circGSK3B, GSK126, and RORA on GC cell proliferative capacity assessed in accordance with cell viability and colony formation. **d**, **e** The impact of circGSK3B, GSK126, and RORA on GC cell invasion and migration capacity evaluated by transwell assay. **f**, **g** The influence of circGSK3B, GSK126, and RORA on GC cell cycle arrest and apoptosis. **h** The effect of circGSK3B, GSK126, and RORA on protein level of PCNA and CD31 in GC cells

We next conducted rescue studies to further confirm the effects circGSK3B, EZH2, and *RORA* on the growth and aggressiveness of GC cells. The results showed that circGSK3B knockdown significantly promoted proliferation, invasion, migration, and cell cycle progression but blocked MKN45 cell apoptosis. This could be rescued by EZH2 knockdown, and this effect was further reversed by *RORA* downregulation (Fig. 6a, c–g; Additional file 3: Figure S2b). Additionally, circGSK3B overexpression significantly suppressed proliferation, invasion, migration, and cell cycle progression, while enhancing AGS cell apoptosis. This could be rescued by high EZH2 expression, and the impact could be further reversed by *RORA* upregulation (Fig. 6b, c–h; Additional file 3: Figure S4). Collectively, these findings indicated that circGSK3B inhibited proliferation, invasion, migration, and cell cycle progression, while enhancing GC cell apoptosis, via the downregulation of EZH2 through the trans-inhibition and upregulation of *RORA* expression.

### Clinical relevance of the circGSK3B/EZH2/RORA axis in GC

**Fig. 7:**
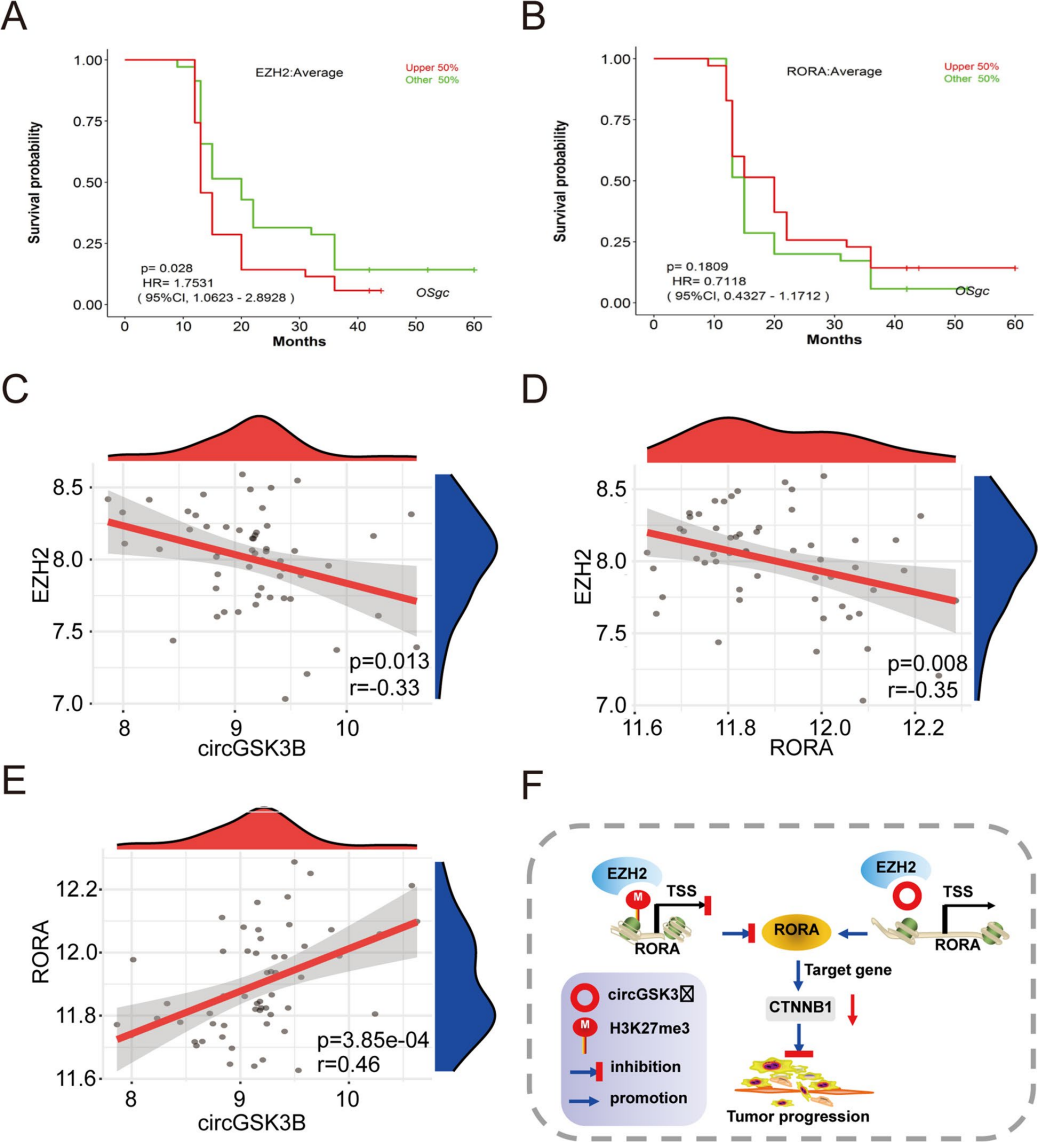
Clinical relevance of the circGSK3B/EZH2/RORA axis in GC. **a** Log-rank test for survival comparison between EZH2 high (*n* = 35) and low group (*n* = 35) in GSE57303. **b** Log-rank test for survival comparison between RORA high (*n* = 35) and low group (*n* = 35) in GSE57303. **c**, **d**, **e** Spearman’s correlation test was performed to verify the association between EZH2, circGSK3B or RORA RNA expression. **F** Schematic diagram of the present study

Next, 56 GC tissues were collected to explore the correlations between the expression levels of circGSK3B, EZH2, and RORA and clinical parameters. RT-qPCR results revealed that low circGSK3B and high EZH2 expression were associated with tumor size (*P* = 0.018 and *P* = 0.018, respectively; Additional file 2: Supplementary Table S7), lymph node metastasis (*P* = 0.029, *P* = 0.006) and TNM stage (*P* = 0.031, *P* = 0.007). Moreover, high EZH2 expression was also correlated with blood vessel invasion (*P* = 0.042) and predicted poor prognosis (Fig. 7a, *P* = 0.028). Meanwhile, low RORA expression was related to TNM stage (*P* = 0.031) and poor prognosis, albeit without statistical significance (Fig. 7b, *P* = 0.18). Notably, the expression levels of circGSK3B (Fig. 7c, *R* = 0.33, *P* = 0.013) or RORA (Fig. 7d, *R* = 0.35, *P* = 0.008) were negatively correlated with those of EZH2 in GC specimens, whereas the expression of circGSK3B was positively correlated with that of RORA (Fig. 7e, *R* = 0.46, *P* < 0.001).

## Discussion

Numerous studies have revealed that circRNAs are closely involved in tumorigenesis. For example, Yao et al. found that hsa_circ_0058124 promotes papillary thyroid cancer tumorigenesis and invasiveness through the NOTCH3/GATAD2A axis [23]. Zhang et al. suggested that circDLST promotes the tumorigenesis and metastasis of GC by sponging miR-502-5p [24]. Cao et al. demonstrated that circRNF20 promotes breast cancer tumorigenesis and the Warburg effect via miR-487a/HIF-1alpha/HK2 [25]. Furthermore, multiple studies have indicated that circRNAs could serve as tumor drivers or inhibitors [26–29]. However, the specific role of circGSK3B in GC and the potential mechanism had not yet been investigated. In our study, we found that circGSK3B levels were significantly decreased in GC samples through the examination of circRNA microarray data from GEO and ArrayExpress databases. We found nine available datasets for potential use in DEC screening. However, to eliminate heterogeneity and increase reliability, we limited our investigation to the six chip datasets, excluding the three sequencing datasets. We combined the *P*-values of these six datasets using the MetaDE package. Fourteen differential circRNAs were identified as exhibiting differential expression in most of these datasets. Further investigation and verification using cell and tissue samples provided evidence suggesting that circGSK3B functions as a tumor driver in GC progression.

We first performed MTT and transwell assays to assess the effect circGSK3B on the two GC cell lines. These results showed that circGSK3B could suppress GC cell proliferation, invasion, and migration. In addition, it is widely accepted that nuclear antigen Ki-67 functions as a cell proliferation hallmark in both clinical carcinoma diagnosis and academic literature [30]. Zhu et al. demonstrated that CD31 and D2-40 contribute to peritoneal metastasis of colorectal cancer by promoting epithelial–mesenchymal transitioning [31], which is consistent with our results presented in Fig. 2f. The outcome of immunohistochemical staining further verified that circGSK3B blocked the growth and invasion of GC cells. The direct binding relationship between circGSK3B and EZH2 was analyzed and verified based on biotin-labeled RNA pull-down, MS, and RIP assays. In addition, the co-localization of circGSK3B and EZH2 in MKN45 and AGS cells was verified by dual RNA-FISH and immunofluorescence assays. Studies have indicated that RORA targets β-catenin directly and modulates canonical Wnt pathway-target genes including, *c-Myc*, *c-Jun*, and *CCND1*. A recent study suggested that miR-129-5p induces cell cycle arrest by modulating *HOXC10*/*CCND1*, in turn inhibiting GC progression. We speculated that circGSK3B could regulate the cell cycle of GC through cyclin D1 [32]. Additionally, a ChIP assay was adopted to explore the regulatory relationship between EZH2/H3K27me3 and *RORA* in GC cells. The results shown in Fig. 5g & h highlight that circGSK3B overexpression significantly increased the *RORA* expression level, which was rescued by EZH2 overexpression. Furthermore, the effect circGSK3B on EZH2, *RORA*, β-catenin, and canonical Wnt pathway-target genes was analyzed by western blot assays and confirmed by a rescue study (Fig. 6).

Numerous researchers have reported that EZH2 might serve as a tumor driver. For example, Chu et al. found that the EZH2-PHACTR2-AS1-ribosome axis could induce genomic instability and promote growth and metastasis in breast cancer [33]. Huang et al. revealed that the inhibition of EZH2 and activation of ERRgamma synergistically suppresses GC via inhibition of the FOXM1 signaling pathway [34]. Wu et al. suggested that EZH2 promoted the expression of LPA1 by mediating microRNA-139 promoter methylation to accelerate the development of ovarian cancer [35], which is consistent with the results of our study. We also verified that EZH2 promoted proliferation, invasion, migration, and cell cycle progression, in addition to blocking apoptosis, in GC cells (Fig. 6). Huang et al. suggested that EZH2 serves as the catalytic subunit of PRC2 and that dysregulation of EZH2 results in a change in gene expression, thus enhancing cancer progression. The canonical function of EZH2 is gene silencing via the catalysis of H3K27me3 trimethylation in a PRC2-dependent manner [36]. The results of our study suggest that circGSK3B functions as a tumor suppressor by inhibiting EZH2-induced histone trimethylation. Furthermore, we truncated EZH2 and explored the binding domain of EZH2 with respect to circGSK3B. This facilitated an exploration of the mechanism in our study.

Various studies have indicated that RORA functions as a tumor suppressor. For example, Sun et al. demonstrated that *RORA* overexpression could rescue the promoting effect of miR-652 on endometrial cancer [37]. Jiang et al. discovered that *RORA* overexpression could inhibit the proliferation and tumorigenesis of glioma cell lines [38]. Zou et al. identified *RORA* as an inhibitor and prognostic biomarker for patients with hepatocellular cancer [39]. The findings of those studies are consistent with our results. Additionally, the results highlighted in Fig. 6 confirm that RORA suppressed the proliferation, invasion, migration, and cell cycle progression, while enhancing apoptosis, in GC cells. Collectively, circGSK3B directly interacts with EZH2 to block the binding of EZH2 to the *RORA* promoter, leading to an increase in RORA expression and the suppression of tumor progression through Wnt signaling pathway regulation. Notably, we also analyzed the relationship between these three molecules and clinical indicators and found that they were indeed associated with some clinical progress indicators, such as tumor size and TNM stage. The prognostic significance of EZH2 and RORA, but not circGSK3B, has also been explored based on the GSE57303 dataset. Further large-scale, well-designed, and multi-center prospective studies should be conducted to confirm our findings before determining whether they are independent prognostic factors or whether they could be combined to predict the prognosis of GC.

## Conclusion

In conclusion, the results of our study suggest that circGSK3B acts as a tumor suppressor. circRNA achieves this effect by reducing EZH2 trans-inhibition, ultimately leading to an increase in *RORA* expression. *RORA* can then suppress tumor progression through regulation of the Wnt signaling pathway. These findings provide a potential basis for the future development of GC treatments.

## Supplementary Information

**Additional file 1: Table S1.** Detailed information of enrolled 6 circRNAs datasets.

**Additional file 2: Table S2.** Oligonucleotide sets used for constructs. **Table S3.** Oligonucleotide sets used for short hairpin RNAs, or probe. **Table S4.** Primer sets used for RT-PCR, qPCR, RIP, and ChIP. **Table S5.** Screening for RBPs and TFs interacting with circGSK3B by MS. **Table S6.** Screening for circGSK3B targets by intersecting DEGs with biogrid targets or tftargets. **Table S7.** Clinical relevance of the circGSK3B/EZH2/RORA axis in GC.

**Additional file 3: Figure S1.** Expression profiles of circRNAs. **(a)** RT-PCR assay with divergent primers showing the detectable (left panel) and undetectable (right panel) circRNAs in cultured MKN45 cells. **(b, c)** The genomic locus of hsa_circ_0036287 (b) and hsa_circ_0000423 (c). The expression level of hsa_circ_0036287 or hsa_circ_0000423 was assessed via RT-PCR assay and Sanger sequencing. Arrows represent divergent primers targeting its genome region. **Figure S2.** Lentivirus-mediated knock down selection and the mass spectrometric results pulled down by the circGSK3B. **(a)** Knockdown efficiency of three sequences targeting circGSK3B. sh-circGSK3B #2 has the highest knockdown efficiency and is used for subsequent experiments. **(b)** Knockdown efficiency of two sequences targeting RORA. sh-RORA #1 has the highest knockdown efficiency and is used for subsequent experiments. **(c)** MS assay showing the EED peptides pulled down by circGSK3B. **(d)** MS assay showing the SUZ12 peptides pulled down by circGSK3B. **Figure S3.** circGSK3B targeted RORA expression through EZH2-mediated epigenetic regulation in GC cells. **(a)** Binding transcription factors to RORA promoter region was assessed by UCSC. (**b**) The effect of circGSK3B overexpression and GSK126 (EZH2 inhibitor) on RORA, H3K27me3, β-catenin, P-β-catenin, and CCND1 (Canonical target gene of β-catenin) assessed by western blot assay in MKN45 and AGS cell lines. (**c, d**) Relative enrichment of EZH2 and its catalytic histone marks H3K27me3 on the promoter region of RORA gene was evaluated by ChIP-qPCR assays in MKN45 and AGS cells. Primer 1 (− 61 ∼ + 45), Primer 2 (− 530 ∼ − 380), Primer3 (− 1019 ∼ − 870), Primer 4 (− 1562 ∼ − 1395) and Primer 5 (− 1973 ∼ − 1731). IgG was used as a negative control. **Figure S4.** Representative images (upper panel) and quantification (lower panel) of immunohistochemical staining showing the expression of Ki-67 and CD31 within xenograft tumors formed by hypodermic injection of MKN45 cells stably transfected with circ-Mock, circGSK3B-OE, circGSK3B-OE + EZH2-OE, circGSK3B-OE + EZH2-OE + RORA+OE.

## Data Availability

Gene expression data (GSE78092, GSE83521, GSE89143, GSE93541, GSE100170, GSE121445, GSE122796, GSE141977, and GSE152309) were downloaded from the Gene Expression Omnibus (http://www.ncbi.nlm.nih.gov/geo).

## Abbreviations

cDNA: Complimentary DNA
ChIP: Chromatin immunoprecipitation
circRNA: Circular RNA
DAPI: 4′,6-diamidino-2-phenylindole
DEC: Differentially expressed circDNA
DEG: Differentially expressed gene
FBS: Fetal bovine serum
FISH: Fluorescence in situ hybridization
GC: Gastric cancer
gDNA: Genomic DNA
GEO: Gene Expression Omnibus
IHC: Immunohistochemistry
RIP: RNA immunoprecipitation
RT-qPCR: Quantitative real-time polymerase chain reaction MS Mass spectrometry
SD: Standard deviation
shRNA: Short hairpin RNA
TF: Transcription factor

## References

[1] Bray F, Ferlay J, Soerjomataram I, Siegel RL, Torre LA, Jemal A (2018). Global cancer statistics 2018: GLOBOCAN estimates of incidence and mortality worldwide for 36 cancers in 185 countries. CA Cancer J Clin.

[2] Moss SF (2017). The clinical evidence linking helicobacter pylori to gastric Cancer. Cell Mol Gastroenterol Hepatol.

[3] Yi H, Qiu MZ, Yuan L, Luo Q, Pan W, Zhou S (2020). Bcl-2/Bcl-xl inhibitor APG-1252-M1 is a promising therapeutic strategy for gastric carcinoma. Cancer Med.

[4] Li X, Yang L, Chen LL (2018). The biogenesis, functions, and challenges of circular RNAs. Mol Cell.

[5] Patop IL, Wüst S, Kadener S (2019). Past, present, and future of circRNAs. EMBO J.

[6] Kristensen LS, Andersen MS, Stagsted LVW, Ebbesen KK, Hansen TB, Kjems J (2019). The biogenesis, biology and characterization of circular RNAs. Nat Rev Genet.

[7] Vo JN, Cieslik M, Zhang Y, Shukla S, Xiao L, Zhang Y (2019). The Landscape of Circular RNA in Cancer. Cell.

[8] Yang H, Zhang H, Yang Y, Wang X, Deng T, Liu R (2020). Hypoxia induced exosomal circRNA promotes metastasis of colorectal Cancer via targeting GEF-H1/RhoA axis. Theranostics..

[9] Xing L, Yang R, Wang X, Zheng X, Yang X, Zhang L (2020). The circRNA circIFI30 promotes progression of triple-negative breast cancer and correlates with prognosis. Aging..

[10] Xie M, Yu T, Jing X, Ma L, Fan Y, Yang F (2020). Exosomal circSHKBP1 promotes gastric cancer progression via regulating the miR-582-3p/HUR/VEGF axis and suppressing HSP90 degradation. Mol Cancer.

[11] Xin D, Xin Z (2020). CircRNA_100782 promotes roliferation and metastasis of gastric cancer by downregulating tumor suppressor gene Rb by adsorbing miR-574-3p in a sponge form. Eur Rev Med Pharmacol Sci.

[12] Peng L, Sang H, Wei S, Li Y, Jin D, Zhu X (2020). circCUL2 regulates gastric cancer malignant transformation and cisplatin resistance by modulating autophagy activation via miR-142-3p/ROCK2. Mol Cancer.

[13] Li K, Cao J, Zhang Z, Chen K, Ma T, Yang W (2020). Circular RNA circGSK3B promotes cell proliferation, migration, and invasion by sponging miR-1265 and regulating CAB39 expression in hepatocellular carcinoma. Front Oncol.

[14] Ramasamy A, Mondry A, Holmes CC, Altman DG (2008). Key issues in conducting a meta-analysis of gene expression microarray datasets. PLoS Med.

[15] Diboun I, Wernisch L, Orengo CA, Koltzenburg M (2006). Microarray analysis after RNA amplification can detect pronounced differences in gene expression using limma. BMC Genomics.

[16] Wang X, Kang DD, Shen K, Song C, Lu S, Chang LC (2012). An R package suite for microarray meta-analysis in quality control, differentially expressed gene analysis and pathway enrichment detection. Bioinformatics (Oxford, England).

[17] Tseng GC, Ghosh D, Feingold E (2012). Comprehensive literature review and statistical considerations for microarray meta-analysis. Nucleic Acids Res.

[18] Lu S, Li J, Song C, Shen K, Tseng GC (2010). Biomarker detection in the integration of multiple multi-class genomic studies. Bioinformatics (Oxford, England).

[19] Lee E, Lee TA, Kim JH, Park A, Ra EA, Kang S (2017). CNBP acts as a key transcriptional regulator of sustained expression of interleukin-6. Nucleic Acids Res.

[20] Jiang C, Xuan Z, Zhao F, Zhang MQ (2007). TRED: a transcriptional regulatory element database, new entries and other development. Nucleic Acids Res.

[21] Cao R, Wang L, Wang H, Xia L, Erdjument-Bromage H, Tempst P (2002). Role of histone H3 lysine 27 methylation in Polycomb-group silencing. Science (New York, NY).

[22] Lee JM, Kim IS, Kim H, Lee JS, Kim K, Yim HY (2010). RORalpha attenuates Wnt/beta-catenin signaling by PKCalpha-dependent phosphorylation in colon cancer. Mol Cell.

[23] Yao Y, Chen X, Yang H, Chen W, Qian Y, Yan Z (2019). Hsa_circ_0058124 promotes papillary thyroid cancer tumorigenesis and invasiveness through the NOTCH3/GATAD2A axis. J Exp Clin Cancer Res.

[24] Zhang J, Hou L, Liang R, Chen X, Zhang R, Chen W (2019). CircDLST promotes the tumorigenesis and metastasis of gastric cancer by sponging miR-502-5p and activating the NRAS/MEK1/ERK1/2 signaling. Mol Cancer.

[25] Cao L, Wang M, Dong Y, Xu B, Chen J, Ding Y (2020). Circular RNA circRNF20 promotes breast cancer tumorigenesis and Warburg effect through miR-487a/HIF-1α/HK2. Cell Death Dis.

[26] Verduci L, Ferraiuolo M, Sacconi A, Ganci F, Vitale J, Colombo T (2017). The oncogenic role of circPVT1 in head and neck squamous cell carcinoma is mediated through the mutant p53/YAP/TEAD transcription-competent complex. Genome Biol.

[27] Wu Z, Sun H, Liu W, Zhu H, Fu J, Yang C (2020). Circ-RPL15: a plasma circular RNA as novel oncogenic driver to promote progression of chronic lymphocytic leukemia. Leukemia..

[28] Li XN, Wang ZJ, Ye CX, Zhao BC, Li ZL, Yang Y (2018). RNA sequencing reveals the expression profiles of circRNA and indicates that circDDX17 acts as a tumor suppressor in colorectal cancer. J Exp Clin Cancer Res.

[29] Xia X, Li X, Li F, Wu X, Zhang M, Zhou H (2019). Correction to: a novel tumor suppressor protein encoded by circular AKT3 RNA inhibits glioblastoma tumorigenicity by competing with active phosphoinositide-dependent Kinase-1. Mol Cancer.

[30] Sales Gil R, Vagnarelli P (2018). Ki-67: more hidden behind a 'Classic proliferation Marker'. Trends Biochem Sci.

[31] Zhu X, Zhou G, Ni P, Jiang X, Huang H, Wu J (2021). CD31 and D2-40 contribute to peritoneal metastasis of colorectal Cancer by promoting epithelial-Mesenchymal transition. Gut Liver.

[32] Huang JL, Fu YP, Gan W, Liu G, Zhou PY, Zhou C (2020). Hepatic stellate cells promote the progression of hepatocellular carcinoma through microRNA-1246-RORα-Wnt/β-catenin axis. Cancer Lett.

[33] Chu W, Zhang X, Qi L, Fu Y, Wang P, Zhao W (2020). The EZH2-PHACTR2-AS1-ribosome Axis induces genomic instability and promotes growth and metastasis in breast Cancer. Cancer Res.

[34] Huang B, Mu P, Yu Y, Zhu W, Jiang T, Deng R (2021). Inhibition of EZH2 and activation of ERRγ synergistically suppresses gastric cancer by inhibiting FOXM1 signaling pathway. Gastric Cancer.

[35] Wu D, Wu F, Li B, Huang W, Wang D (2020). EZH2 promotes the expression of LPA1 by mediating microRNA-139 promoter methylation to accelerate the development of ovarian cancer. Cancer Cell Int.

[36] Huang J, Gou H, Yao J, Yi K, Jin Z, Matsuoka M (2021). The noncanonical role of EZH2 in cancer. Cancer Sci.

[37] Sun X, Dongol S, Qiu C, Xu Y, Sun C, Zhang Z (2018). miR-652 promotes tumor proliferation and metastasis by targeting RORA in endometrial Cancer. Mol Cancer Res.

[38] Jiang Y, Zhou J, Zhao J, Hou D, Zhang H, Li L (2020). MiR-18a-downregulated RORA inhibits the proliferation and tumorigenesis of glioma using the TNF-α-mediated NF-κB signaling pathway. EBioMedicine..

[39] Zou Y, Sun H, Guo Y, Shi Y, Jiang Z, Huang J (2021). Integrative Pan-Cancer analysis reveals decreased Melatonergic gene expression in carcinogenesis and RORA as a prognostic marker for hepatocellular carcinoma. Front Oncol.

