# Supplementary material for: CircGSK3B promotes RORA expression and suppresses gastric cancer progression through the prevention of EZH2 trans-inhibition
Source: J Exp Clin Cancer Res. 2021 Oct 19;40:330. doi: 10.1186/s13046-021-02136-w (PMC8524915; doi:10.1186/s13046-021-02136-w)
Supplement: Supplementary file 2 — Additional file 2: Table S2. Oligonucleotide sets used for constructs. Table S3. Oligonucleotide sets used for short hairpin RNAs, or probe. Table S4. Primer sets used for RT-PCR, qPCR, RIP, and ChIP. Table S5. Screening for RBPs and TFs interacting with circGSK3B by MS. Table S6. Screening for circGSK3B targets by intersecting DEGs with biogrid targets or tftargets. Table S7. Clinical relevance of the circGSK3B/EZH2/RORA axis in GC. [file 13046_2021_2136_MOESM2_ESM.docx]

**Supplementary Table S2 Oligonucleotide sets used for constructs**

| **Oligo Set** | **Sequences** |
| --- | --- |
| pLCDH-circGSK3B | 5'-CCGGAATTCTGAAATATGCTATCTTACAGGTCTTCCGACCCCGAACTCCAC-3' (sense) |
|  | 5'-CGCGGATCCTCAAGAAAAAATATATTCACCTGACGCTGCTGTGGCATTTGTG-3' (antisense) |
| pLCDH-linGSK3B | 5'-CCGGAATTCTGAAATATGCTATCTTACAGGTCTTCCGACCCCGAACTCCAC-3' (sense) |
|  | 5'-CGCGGATCCGGCTGACGCTGCTGTGGCATTTGTG-3' (antisense) |
| pCMV-3Tag-1A-EZH2  (1-751) | 5’-CGCGGATCCATGGGCCAGACTGGGAAGAAATC-3’ (sense); |
|  | 5’-GCCGCTCGAGAGGGATTTCCATTTCTCTTTCGA-3’ (antisense) |
| pCMV-3Tag-1A-EZH2  (1-618) | 5’-CGCGGATCCATGGGCCAGACTGGGAAGAAATC-3’ (sense); |
|  | 5’-GCCGCTCGAGATGCTTTTTGGAGCCCCGCTGAA-3’ (antisense) |
| pCMV-3Tag-1A-EZH2  (1-350) | 5’-CGCGGATCCATGGGCCAGACTGGGAAGAAATC-3’ |
|  | 5’-GCCGCTCGAGGGTCTTTATCCGCTCAGCGGTGA-3’ (antisense) |
| pCMV-3Tag-1A-EZH2  (1-180) | 5’-CGCGGATCCATGGGCCAGACTGGGAAGAAATC-3’ (sense); |
|  | 5’-GCCGCTCGAGTTGACCAAGGGCATTCACCAACT-3’ (antisense) |
| pCMV-3Tag-1A-EZH2  (200-350) | 5’-CGCGGATCCCAGAAAGATCTGGAGGATCACCG-3’ (sense); |
|  | 5’-GCCGCTCGAGGGTCTTTATCCGCTCAGCGGTGA-3’ (antisense) |
| pGEX-6P-1-EZH2  (1-751) | 5’- CGCGGATCCATGGGCCAGACTGGGAAGAAATC -3’ (sense); |
|  | 5’- GCCGCTCGAGAGGGATTTCCATTTCTCTTTCGA -3’ (antisense) |
| pGEX-6P-1-EZH2  (1-618) | 5'- CGCGGATCCATGGGCCAGACTGGGAAGAAATC -3’ (sense); |
|  | 5'- GCCGCTCGAGATGCTTTTTGGAGCCCCGCTGAA -3’ (antisense) |
| pGEX-6P-1-EZH2  (1-350) | 5’- CGCGGATCCATGGGCCAGACTGGGAAGAAATC -3' (sense); |
|  | 5'- GCCGCTCGAGGGTCTTTATCCGCTCAGCGGTGA -3’ (antisense) |
| pGEX-6P-1-EZH2  (1-180) | 5’- CGCGGATCCATGGGCCAGACTGGGAAGAAATC -3' (sense); |
|  | 5’- GCCGCTCGAGTTGACCAAGGGCATTCACCAACT -3' (antisense) |
| pGEX-6P-1-EZH2  (200-350) | 5’- CGCGGATCCCAGAAAGATCTGGAGGATCACCG -3’ (sense); |
|  | 5’- GCCGCTCGAGGGTCTTTATCCGCTCAGCGGTGA -3' (antisense) |
| pCMV-3Tag-1A-RORA | 5’- CGCGGATCCATGGAGTCAGCTCCGGCAGCCC -3’ (sense); |
|  | 5’- GCCGCTCGAGCCCATCAATTTGCATTGCTGGCT -3’ (antisense) |

**Supplementary Table S3 Oligonucleotide sets used for short hairpin RNAs, or probe**

| **Oligo Set** | **Sequences** |
| --- | --- |
| sh-Scb | 5'-CCGGGCGAACGATCGAGTAAACGGACTCGAGTCCGTTTACTCGATCGTTCGCTTTTT-3' (sense); |
|  | 5'-AATTCAAAAAGCGAACGATCGAGTAAACGGACTCGAGTCCGTTTACTCGATCGTTCGC-3' (antisense) |
| sh-circ-GSK3B #1 | 5'-CCGGGCAGCGTCAGGTCTTCCGACCCTCGAGGGTCGGAAGACCTGACGCTGCTTTTTG-3' (sense); |
|  | 5'-AATTCAAAAAGCAGCGTCAGGTCTTCCGACCCTCGAGGGTCGGAAGACCTGACGCTGC-3' (antisense) |
| sh-circ-GSK3B #2 | 5'-CCGGAGCGTCAGGTCTTCCGACCCCCTCGAGGGGGTCGGAAGACCTGACGCTTTTTTG-3' (sense); |
|  | 5'-AATTCAAAAAAGCGTCAGGTCTTCCGACCCCCTCGAGGGGGTCGGAAGACCTGACGCT-3' (antisense) |
| sh-circ-GSK3B #3 | 5'-CCGGCCACAGCAGCGTCAGGTCTTCCTCGAGGAAGACCTGACGCTGCTGTGGTTTTTG-3' (sense); |
|  | 5'-AATTCAAAAACCACAGCAGCGTCAGGTCTTCCTCGAGGAAGACCTGACGCTGCTGTGG-3' (antisense) |
| sh-RORA #1 | 5'-CCGGGCTGTTCTGGTCTCAACTTGACTCGAGCGACAAGACCAGAGTTGAACTTTTTTG-3' (sense); |
|  | 5'-AATTCAAAAAGCTGTTCTGGTCTCAACTTGACTCGAGCGACAAGACCAGAGTTGAACT-3' (antisense) |
| sh-RORA #2 | 5'-CCGGGGAGAAGTCAGCAAAGCAATGCTCGAGCATTGCTTTGCTGACTTCTCCTTTTTG-3' (sense); |
|  | 5'-AATTCAAAAAGGAGAAGTCAGCAAAGCAATGCTCGAGCATTGCTTTGCTGACTTCTCC-3' (antisense) |
| circ-GSK3B probe | CCACAGCAGCGTCAGGTCTTCCGACCCCGA (antisense)  GGTGTCGTCGCAGTCCAGAAGGCTGGGGCT (sense) |

**Supplementary Table S4 Primer sets used for RT-PCR, qPCR, RIP, and ChIP**

| **Primer set** | **Primers** | **Sequence** | **Product size (bp)** | **Application** | |
| --- | --- | --- | --- | --- | --- |
| hsa_circ_0036287 | Forward | 5’- GAGGACTACTATGGACCTTCACG -3' | 226 | RT-PCR, qPCR | |
|  | Reverse | 5’- TCCCTTCATGTTCCCGACT -3' |  |  |  |
| hsa_circ_0000423 | Forward | 5’- AGGGAGACTGTGACCTACT -3' | 426 | RT-PCR, qPCR | |
|  | Reverse | 5’- TGCCATTGGCTCTGCATTT -3' |  |  |  |
| hsa_circ_0000554 | Forward | 5’- ATCCATCGCCGTCTTCAGC -3' | 156 | RT-PCR | |
|  | Reverse | 5’- GGCTCTTACCGTCTGTGGG -3' |  |  |  |
| hsa_circ_0007991 | Forward | 5’- CCCTCAAACCCCTCAGACAA -3’ | 141 | RT-PCR | |
|  | Reverse | 5’- TTGCCACTTGTGTTACCGTG -3' |  |  |  |
| hsa_circ_0001380 | Forward | 5’- CGGCCACCCATTGATTTGAT -3' | 126 | RT-PCR | |
|  | Reverse | 5’- CGTCGTCTTTTAGGAGCACC -3' |  |  |  |
| hsa_circ_0092337 | Forward | 5’- TTCCATTGACGTTGCTCTCC -3' | 248 | RT-PCR | |
|  | Reverse | 5’- TCTTCTCACCCCAGCAAGAC -3' |  |  |  |
| hsa_circ_0007376 | Forward | 5’- GTGACTTCGGGGTGAGCGG -3' | 153 | RT-PCR | |
|  | Reverse | 5’- TCACGAGGATGTTGGAGGG -3' |  |  |  |
| hsa_circ_0009594 | Forward | 5’- AGCCACCACAGCATCTTTC -3' | 127 | RT-PCR | |
|  | Reverse | 5’- GGAGTTGTGGACCAGTTTG -3' |  |  |  |
| hsa_circ_0013048 | Forward | 5’- GTCGGACGGATGACAAGAT -3' | 285 | RT-PCR | |
|  | Reverse | 5’- AAGCTGCTCTGCTGAAACC -3' |  |  |  |
| hsa_circ_0048607 | Forward | 5’- ACTCTGTACTCAGCCATTCGT -3' | 296 | RT-PCR | |
|  | Reverse | 5’- CAAGTTTCGGTCTAAAGTCTATG -3’ |  |  |  |
| hsa_circ_0067934 | Forward | 5’- CTTCCGAAATGTTGATTG -3' | 117 | RT-PCR | |
|  | Reverse | 5’- TTCCTTAGGGTCCTTATT -3' |  |  |  |
| hsa_circ_0005397 | Forward | 5’- TCTGTATAGTGTATGCCGTTAA -3' | 106 | RT-PCR | |
|  | Reverse | 5’- GGGAGGAACCTGCTGTCT -3' |  |  |  |
| hsa_circ_0005051 | Forward | 5’- GGCAGCTTTGAAACACTC -3' | 98 | RT-PCR | |
|  | Reverse | 5’- TTCTTGTTCTTGCCGAAT -3' |  |  |  |
| CircGSK3B  (Divergent) | Forward | 5'- CCATCCTTATTCCTCCTCA -3’ | 148 | RT-PCR,  qPCR, RIP | |
|  | Reverse | 5'- GTCGGGCAGTTGGTGTAT -3’ |  |  |  |
| circGSK3B  (Convergent) | Forward | 5'- TACACCAACTGCCCGACTA -3’ | 180 | | RT-PCR |
|  | Reverse | 5'- CGAGCATGAGGAGGAATAAG -3’ |  |  |  |
| GAPDH | Forward | 5'- AGAAGGCTGGGGCTCATTTG -3' | 258 | | RT-PCR, qPCR |
|  | Reverse | 5'- AGGGGCCATCCACAGTCTTC -3' |  |  |  |
| RORA | Forward | 5'- ACTCCTGTCCTCGTCAGAAGA -3' | 95 | | qRT-PCR |
|  | Reverse | 5'- CATCCCTACGGCAAGGCATTT -3' |  |  |  |
| GNG13 | Forward | 5'- ACCATCCCCGAGCTGCTGA -3' | 104 | | qRT-PCR |
|  | Reverse | 5'- CATTTGCCCTTTTCCACCC -3' |  |  |  |
| MTHFD2 | Forward | 5'- TGGCTGCGACTTCTCTAATG -3' | 128 | | qRT-PCR |
|  | Reverse | 5'- CCTTCCAGAAATGACAACAGC -3' |  |  |  |
| CDKN1A | Forward | 5'- CCCGTGAGCGATGGAACTT -3’ | 317 | | qPCR |
|  | Reverse | 5'- GAAATCTGTCATGCTGGTCTGC -3’ |  |  |  |
| CDKN2B | Forward | 5'- CGGCAGCGATGAGGGTCTG -3' | 110 | | qPCR |
|  | Reverse | 5'- GCCTCCCGAAACGGTTGACT -3' |  |  |  |
| FHOD3 | Forward | 5'- GACTGGAGCACCTGTTTGA -3' | 233 | | qPCR |
|  | Reverse | 5'- GGTGGGAATCATCGTTAGAAT -3' |  |  |  |
| FBXO32 | Forward | 5'- AAGTCTGTGCTGGTCGGGAA -3' | 123 | | qPCR |
|  | Reverse | 5'- AGTGAAGGTGAGGCCTTTGAAG -3' |  |  |  |
| PHGDH | Forward | 5'- TACCTCTGCTCTAACTCCTCCCT -3' | 179 | | qPCR |
|  | Reverse | 5'- CATTTGCCGTCCTTCATCG -3' |  |  |  |
| TWIST1 | Forward | 5'- GCCGACGACAGCCTGAGCAA -3' | 210 | | qPCR |
|  | Reverse | 5'- GCCACAGCCCGCAGACTTCTT -3' |  |  |  |
| PTPN12 | Forward | 5'- AGCGGGAGGTATTCACTAT -3' | 139 | | qPCR |
|  | Reverse | 5'- TCTTGGTCCTTTGGGTTTT -3' |  | |  |
| RORA promoter  Primer1(-61/+45) | Forward | 5'- CGGTCGCCTTCTCCTTCTT -3' | 106 | | ChIP |
|  | Reverse | 5'- CCGAGACTCCCTCTATCTT -3' |  |  |  |
| RORA promoter | Forward | 5'- AGTTTCTCCTCAGCCCTCG -3' | 151 | | ChIP |
| Primer2 (-530/-380) | Reverse | 5'- TGATTCCGTGCCTACTTCT -3' |  | |  |
| RORA promoter | Forward | 5'- GAGGGTGTAGAATGATGCC -3' | 150 | | ChIP |
| Primer3 (-1019/-870) | Reverse | 5'- CAATTCCCACTGGTACTTTT -3' |  | |  |
| RORA promoter | Forward | 5'- GAAGAGTGGACCACAGGTT -3' | 168 | | ChIP |
| Primer4(-1562/-1395) | Reverse | 5'- AATAGTAAGTGCGGGAAAC -3' |  | |  |
| RORA promoter  Primer5(-1973/-1731) | Forward | 5'- CACTTGCGTCCCAGATAGA -3' | 243 | | ChIP |
|  | Reverse | 5'- TAGCCTTCACAACATTTACC -3' |  |  |  |
|  |  |  |  | |  |

**Supplementary Table S5. Screening for RBPs and TFs interacting with circGSK3B by MS**

| MS | TFs | RBPs | Overlap |
| --- | --- | --- | --- |
| CSPG4, GOT2, MACF1, ERAP1, ACOT7, UNC13D, PRPF6, NCEH1, PAPSS1, MYG1, CD81, LRPAP1, COASY, MAPK1, PQBP1, TERF2IP, RBM12B, GOLT1B, SLC30A1, PPAN, RHEB, DDX27, SEH1L, EEF1A2, MRPL43, MVP, FARP1, THOC1, STX4, NCK1, MMAB, AGA, HGS, SNAP23, OTUD5, GPAA1, PON2, APMAP, WDR70, OAS3, DHRS1, MCTS2P, LPGAT1, SIN3A, RGS10, RPL17, SCYL1, KYNU, USP48, S100A4, ALDH6A1, POGZ, NAGA, PRKCSH, GGT1, DNAJC13, TMED6, PGM2, ACO1, FXR2, DYNC1I2, VPS29, RRP12, ATP5F1D, STXBP2, SUN1, ZADH2, GTPBP6, PDE3A, NQO2, SMPD4, HTATIP2, UGP2, NCLN, PTPRF, UIMC1, TOX4, CDC42, PTPN23, RNPEP, HIGD2A, ANKHD1, PPP1R8, GNAI2, CYB5A, LETMD1, FAR1, GBF1, NADK2, TMX4, HSF1, NOC3L, GANAB, GDI1, STAT1, EIF3D, GRIPAP1, DCAF1, SMCHD1, GTF3C4, SUGT1, PBX1, SEPHS1, USP24, HK2, ZHX1, MOV10, MAVS, NUP85, NIF3L1, DNAJC5, THOC5, SEC23B, PLRG1, VMP1, DHX57, PLOD3, CLUH, PALMD, RAB34, SMN1, FRG1, NDUFA9, RBM22, ACAT2, TBL3, AK4, DMAC1, CYB5R1, POLA1, SMARCA2, OGFOD1, NDUFS5, PNP, PDE12, IDE, HMGCS1, SELENOF, ACBD5, TBC1D15, CNP, MRPS5, PARP9, PAK2, DDAH1, MTARC1, RAB2A, PDPR, VWA8, LRP1, MIA3, BID, SLC12A2, NUP205, PTRH2, POLD3, C8orf82, ASF1B, PCYOX1, RPP40, HSPE1-MOB4, MITD1, STX6, SMARCAD1, IL18, FANCI, HSPA14, GNAI1, NNMT, CS, EMC3, SACM1L, AKAP1, FDPS, EDC4, EXOC4, PREP, PREPL, ARIH1, OARD1, PAFAH1B1, RNF20, MRPL13, DNPEP, NFIB, TSG101, UNC93B1, OGA, ITPR1, SAMHD1, PPFIBP1, HIDE1, SPAG9, CYB5R3, SLC35F2, ABCB6, MRPL9, SEC24A, KRTCAP2, AFG3L2, UAP1, MAOB, CRLF3, GBA, IST1, HDAC5, BORCS6, IMP4, , DCAF7, PSMG2, GOLM1, STAT2, PEX19, TSC22D1, STX12, JMJD6, DNAJB4, PDE4D, DENR, SYNE1, C1orf52, GNAQ, HSPBP1, ROCK2, IDH3A, PELP1, FKBP14, WRNIP1, EIF2B5, SCCPDH, TMX2, COQ6, RTF1, RRM1, FABP5, BRIX1, ECH1, COA6, EPHX1, PBRM1, SCARA5, PMF1-BGLAP, AGO1, ALDH1L2, UCHL3, PIK3C3, DDX54, TMF1, PFN2, DCTN1, FXN, NUDT9, MRPS28, MAD1L1, CYFIP1, MAN2A1, TLE1, AKR1A1, SLC33A1, SUDS3, NDUFB10, RAB27B, MED15, DHX8, NSF, BCS1L, UBE2T, YTHDC1, NIT2, CDK5RAP3, LNPEP, CD99, HFE, DDOST, PPIC, PSMB9, DLST, REEP6, SNX4, AP2A2, RPP38, MTERF3, PSMA1, ARF5, SUCLA2, RPL7L1, TBCA, AGL, STIM1, CTSB, CISD2, USP47, TMX3, IBA57, UQCC2, TWF1, HSD17B8, ATP5PB, EED, DNAAF5, GTPBP10, CRYBG2, HEATR1, FECH, MACO1, DAXX, SRP68, TTC37, VPS13A, ZMYM2, COLGALT1, SELENOI, RAMACL, FKBP8, GSK3B, CDK12, DNAJC10, FSTL1, PCBP2, GSS, UBE2Z, DHX29, PSMB8, DHCR24, FLOT1, TK1, MECR, GSPT1, GOSR1, GBE1, EEFSEC, XPOT, HMOX1, COQ9, SUPT5H, FKBP5, DDX28, NOL6, TCEA1, UBL7, QDPR, TRIP13, NDUFS7, ETF1, SMIM20, MRPL45, CSF2RA, RPL30, RPL35, OTUB1, NFRKB, NCOA5, ECHDC3, GTF2F2, NAA15, SP2, TRIM33, UBA6, DDX20, NAP1L1, EXOSC6, CAAP1, SLC4A7, TM9SF2, PLXND1, VAT1, PSMB10, RO60, HIBCH, PPIL2, CARS2, TUBB8, BLMH, RNF40, GOLGA3, SNRNP70, PRKAA1, TPT1, GCSH, STAG2, KIFBP, UBE3A, NR2C2, CHAMP1, BAK1, CNOT2, TOPBP1, ATP5PF, AUP1, MNAT1, ACLY, GANAB, SLC39A14, INTS13, PFDN6, SEC23A, IQGAP2, POLR3B, SNTB2, PLPBP, APEH, MAP7D1, TKT, SLK, MRPS30, GARS1, HAGH, TASOR, SEPTIN6, BAG6, HDHD5, MED16, RHOB, DHODH, MRPL52, PHIP, SURF1, PNPLA4, WDR75, DCTN4, NOL3, SERPINB6, SLC7A1, LRRC41, MARS1, UFSP2, BUB1B, NQO1, PITRM1, RAB8A, ZBTB7B, MED24, CDC27, CUTA, CDC16, AK2, GOLGB1, DDX51, RTN3, ANXA9, RBM12, GSE1, BROX, NUP88, KDM3B, FERMT2, ARHGDIA, PSMA7, SCAF1, PMM2, NBAS, TECR, ARMC10, NVL, POR, SUPV3L1, PELO, NKRF, UBLCP1, EXOSC9, BRCC3, RSL1D1, CAP1, SCAMP4, ACSL4, LSS, VASN, AARS2, NENF, PRMT5, SRPK1, ACO2, PTPN1, MAN1B1, METAP1, GNPDA1, L2HGDH, PFDN1, SCARB1, ANKMY2, TIMP1, AGTR1, INSR, STX3, RIN1, UNC45A, PSMB7, PSMD3, PPOX, SAMD9, TSFM, MRM3, JAK1, ATM, AKR7A2, CHCHD10, SLC25A12, PRKACA, FARSA, PRAF2, GCDH, CCDC58, MRPL4, QARS1, NDUFA3, ACAD9, VIRMA, TACC1, GNL1, EIF1B, SUCLG2, MESD, BLOC1S6, ZMYND8, MEN1, YIPF2, NAMPT, NEPRO, HAX1, TIMM22, ZNF534, NSDHL, GTF2H4, SLC38A2, URB2, DEPDC7, SEC22B, ORC3, SEC24C, LARS1, MRPL15, CNOT1, PLBD2, RRAGC, TBCD, COG3, RECQL, TRMT1, LPP, CEPT1, GLA, PPP1R7, NUP155, TOR4A, UBR7, PUS1, SSR3, GOLM2, CTR9, TCIRG1, TOM1, DCPS, GDI2, CNTNAP1, , ESYT2, NLE1, NCAPD3, DECR1, ATP2C1, IPO11, NACC1, ANPEP, ETFB, KMT2A, CCNK, ANO6, RBM6, U2SURP, PPP2R5E, UBE2A, PRDX2, CHPT1, CCT7, HSPA4L, LMAN1, ETHE1, NOP56, RPL32, RAP2C, PRPSAP1, NUTF2, DCK, PFN1, PMS2, CCAR2, ST20-MTHFS, SDHA, PICALM, MCEE, SFSWAP, SART3, CLASP1, BABAM2, MTHFD1L, SERPINB8, ARID1A, CTPS2, MMUT, CCNB1, FYCO1, CSK, GALNT7, LRCH4, ALDH7A1, SEPTIN8, HMBS, GYS1, TRIP12, CCNA2, EXOSC5, PDCD5, FLAD1, SLC27A2, PAK1IP1, PTK7, FKBP4, ITGB4, YJU2, UGGT1, SNRPA, SFXN1, AKAP12, KIF21A, PEX3, PLLP, MAT2B, PODXL2, PRDX3, GLOD4, SMARCA4, MAGT1, RIF1, ARFGAP1, AGPAT5, NUCB2, CLPTM1L, ERO1A, RPS16, PGRMC2, SRRM2, GTPBP3, MTA2, RHOT2, TMEM11, NARS2, UBR2, METTL9, KATNAL2, HSD17B12, PRKACB, PTPA, PCIF1, GOLIM4, TOLLIP, CNDP2, DUSP12, UFL1, GOLGA5, MRPL37, MLH1, MCAM, EIF2B3, ADSS2, NCKAP1, NUP210, POLE, NANS, AASDHPPT, MRPS10, NT5C2, MTIF2, CARS1, MFSD10, ATPAF1, ATP13A1, VPS26A, RBPMS, NAPRT, NAA80, CARD19, WDR48, BET1L, LRPPRC, HLA-C, TMX1, MCU, SNX6, CDK5RAP1, STAU1, GSPT2, PIH1D1, INTS2, C12orf43, MGST2, GGCX, BAZ1A, PIK3R4, DDT, CSTB, FOSL1, GALM, COPB1, TSTA3, STT3B, TBL2, FKBP10, IKBKG, ZMYM3, COPA, NUP98, ZPR1, NAXE, TAPBPL, KYAT1, MDH1, ALDH2, DGKA, TARS1, PPAT, RAB10, RGPD5, ATP6V0A2, NOP58, ESYT1, PPFIA1, BYSL, ECPAS, IRF2BP2, VWA5A, PPP6R3, TIPRL, ECI1, TOE1, SRP54, LGALS3BP, RPN2, COPS4, IARS1, COPS3, NSUN2, ATAD2, GLUD1, TFRC, DDX23, PEX11B, UBA1, HSD17B4, INTS1, COPB2, ZNF768, HEXB, MTMR2, EEA1, TPI1, ESD, AXL, PLA2G4A, CHST3, AHCYL1, OSGEPL1, YWHAH, ATP6V0A1, SUZ12, ASPM, PRKDC, EIF4B, EIF3L, ADNP, PAICS, ARAF, LONP1, SCP2, CENPB, OGFR, SDF4, AP3B1, IFITM3, ATP1B3, LZTFL1, NPEPPS, PUM1, GSTM1, NUDC, AKAP13, CORO1B, ANXA8L1, BTAF1, RRP8, PSMC1, TARS2, TFG, PGD, RETREG3, URB1, PTPMT1, VPS35L, ARNT, AP1B1, CUL2, ECSIT, IDH3G, VAMP3, ARL6IP5, AKR1C3, LGALS8, ARPP19, SVIL, EIF4A2, BTF3L4, RANBP1, RPL14, DIAPH3, RDX, SERPINB1, DLD, PTCD3, HDAC3, POP1, TRNT1, GPN1, NCCRP1, TRIM47, SIRT1, CUL3, TIMM44, PSMD12, CNPY3, CCDC22, CYP51A1, MGAT1, ENOPH1, NUDT16L1, GNS, RPL3, DTX3L, PISD, CADM4, PSAP, RPN1, CFDP1, BCAT2, PPIL4, CYC1, BMS1, GSTM3, HSP90B1, RPS15A, SLC16A3, BAZ1B, SH3BGRL, BLOC1S3, CTSD, GAPVD1, ACAP2, SKP1, SLC25A3, RNF113A, GALK1, NAA50, CAT, CDKAL1, INO80E, RPL4, FADS3, RNH1, PEBP1, SMC3, CEP72, MSN, ACADVL, COBL, GNPNAT1, COL7A1, C1orf226, TMEM41B, TRAP1, CIAPIN1, PGM1, SFXN3, KANK1, ATRIP, PSMD6, USP34, KDM2A, SLC35A2, COX6A1, KIF4A, PPIG, KDM1A, ROBO1, CUL5, CANX, HKDC1, PHAX, STRBP, TMTC3, LIG1, UBXN7, TPMT, NUP160, NRDC, MBLAC2, PRDX5, NCOR1, DPP3, ELAC2, COG5, NOMO1, ATR, PFKFB2, BAG1, MPHOSPH8, PGP, DDX39A, STT3A, VAV2, RCN3, SHTN1, ZMPSTE24, HSPA4, USP7, L1CAM, H3C15, TEX10, SLC25A1, MRPL28, EPS15, DPM1, TNPO3, SLC7A5, UBAC2, DARS1, SLC4A2, STMN1, ZNF622, ASNS, EIF2A, ERCC6, TXLNG, DDX50, MALSU1, NXN, CXADR, ATP1A1, IARS2, RANBP6, NSMCE1, ARSL, DYNC1H1, COX7A2L, SAMD1, DDX24, VCP, MCAT, , CCT4, P4HB, ENG, EHD1, RPL26, SMS, SOD2, PIK3C2A, ECHS1, ABHD14B, TMSB4X, TMBIM1, SRRM1, PAXIP1, TMSB10, GPS1, FBL, ALDH4A1, OSBPL10, AKIRIN2, POLR2B, SETD3, CASC3, ABCF2, ADK, GALNT1, SNX9, RPL13A, ITCH, RARS1, MARCKS, SEC11A, TES, POLR3A, ERGIC2, SEC24B, RPS8, PSMC3IP, PRKAR1A, RAN, SMARCA5, IGFBP7, TMEM87A, ACBD6, ENO3, EXOC5, DLAT, LCP1, PGK1, PPA2, EEF1D, AGK, TXNDC12, ME1, RPS23, VMA21, NACA, NUCB1, TRIM24, RAB3GAP2, NDUFAF4, STAU2, RETSAT, PYCR2, PCSK9, PSAT1, DCUN1D1, TMED4, ERCC5, C9orf40, RCOR3, SEC62, ZKSCAN1, VKORC1L1, CASP9, NF2, NAT10, TBRG4, OXSM, PFDN2, RPS6KA3, UPF2, AP3D1, SPR, , POLG, ACP6, NUP93, SCO2, GCN1, ACTR5, PKP4, EIF3B, DCUN1D5, WDR77, IPO4, NASP, HADHA, NKAP, KLHL13, PTGR1, GAPDH, PITX1, CAND1, CEBPG, RPS2, PKMYT1, UQCRB, TOMM70, ERP29, PRPF4B, LYN, GPD2, RPL5, VPS33B, LAP3, SYPL1, UBE4B, RFC1, SLC25A13, PCYT1A, MYOF, GGH, EEF2, CSRP1, PDIA4, COPS8, PROCR, TIMM9, SRSF2, SLC25A5, CSE1L, MECOM, RCN1, PDIA3, PSME3, EXOC7, ALDOA, STIP1, BLVRA, LLGL2, QPCTL, CDK11A, LPCAT4, UBXN4, PSMG1, RBM47, AP2B1, CARMIL1, MCM5, PSMF1, TMEM214, ANXA5, RPL6, DAP3, DDX18, GSTP1, USP9X, HAT1, TMED7, ARL15, EIPR1, DHX40, REEP3, PDXDC1, UBFD1, ALDH18A1, H1-3, NDUFS2, BCCIP, HAUS5, ITPRID2, LSM8, SNRPC, FH, CHD2, ACOT9, ARMT1, CCT8, SFN, DNPH1, HNRNPK, , DAD1, ITGA1, PMPCA, PCBD1, DLG1, CLTC, FOSL2, MPST, CDC42BPB, NUDT5, DCTN2, DNMT1, PSME2, UBE2K, CUL1, PRPF19, NCAPG2, DDX47, AP1G1, NRAS, APOC3, MAN2B1, GET3, SCAMP3, SARS1, RAB14, TPM2, POLDIP2, ZBTB45, LAMB1, RBBP7, SCRN1, GTPBP4, NPLOC4, AGMAT, MCFD2, SLC9A3R2, YWHAB, CMC1, GEMIN5, VCL, H6PD, HARS1, PFAS, GIPC3, RPS27, PPP2CA, XRCC5, MTF2, DDX39B, MBD4, PLS3, PPIP5K2, GNAI3, CDK1, UCHL5, NOTCH2, KIF2A, LPCAT1, EIF1AD, PPT1, ANAPC5, ALDH3A2, GNL3L, SRPRA, EXOSC8, LAS1L, RALY, ABCD3, ABCB10, BCOR, HSP90AA1, RAB1A, GPX8, SELENBP1, RPSA, ARL1, VLDLR, RPL7A, PMPCB, STOM, DHX30, DPP7, SNX3, MRI1, MED23, YARS2, KPNA4, GLT8D1, KHDRBS1, , SMARCE1, TRIP6, TPP2, SLC9A3R1, LEMD2, RAC1, AIFM1, ACACA, WDR36, RMDN1, PRPF8, MTOR, TMEM126A, CAPN2, MRFAP1, PGAM1, SYF2, EPS15L1, NPNT, EZR, BCAM, USO1, BRAT1, DIS3, FANCD2, AARS1, MDN1, PLIN3, HADHB, TMED5, PDCD4, ATAD3A, PLEKHG4, GRB2, CHTOP, MRPS36, TRIM11, SLC38A5, SNX2, PARP14, ING1, NIP7, HSPH1, RPP30, EXOSC7, AQR, STK4, MYBBP1A, UQCRC1, SMC5, SRI, WDR26, HPDL, EFTUD2, IMPDH2, IGF2R, H2AC14, MRPL39, MED22, ALDH3A1, PFKP, H1-1, TPR, TMEM165, ALCAM, VAMP8, HIBADH, CKAP5, CTPS1, RPS6, MCTP2, FAM162A, RPL18A, PAPSS2, EZH2, CLPP, PDS5B, NDRG3, WASHC4, WDR33, MRPS17, PLXNB2, FOXK2, ZC3H11A, PAF1, UTP15, PPP4R3B, CCAR1, RAB5A, MFN2, SRSF1, OAT, ATP5PD, FAM174A, EDF1, KHSRP, ACAA2, CRK, APRT, MRPL21, BSG, PDHA1, EFCAB14, EIF3C, NDC80, XAB2, ADGRE5, CDKN1C, CDK6, RPL13, HS1BP3, HNRNPUL1, PDHB, HEXIM1, NIBAN2, DUT, MSH2, ITSN2, GMPS, SLC25A20, DYNC1LI2, SLC4A1AP, RPL8, EXOSC1, PPDPF, GET4, TBC1D13, TUBGCP2, PKN2, TPRN, HSPG2, INTS11, UBA2, NUP43, TRIM28, CPSF2, YWHAZ, IWS1, CWC27, AP2A1, YWHAQ, SLC25A11, CRNKL1, ETFA, EIF2B4, SRPK2, ACSF3, POLR2L, RAB21, CAVIN3, ADCY9, GCC2, LDLR, UBE2M, CYFIP2, FAHD1, CPSF7, NDUFB4, DOCK5, RABEP2, STUB1, SNRPN, DDX42, MCM2, PACSIN2, AHCY, CPT1A, HLA-A, PSMB4, ANKFY1, ANAPC7, ANXA3, PUS7, HINT1, SOD1, TUBA4A, COPZ1, PNPT1, HLA-C, ACOX1, MLEC, RAB35, ITGB1, ST13, APOO, ERP44, TSN, ISY1, SQOR, CSTF1, IDH1, TXNRD1, HDAC1, HPCAL1, XPNPEP1, FTH1, PRXL2A, SF3B1, IER3IP1, SGPL1, GORASP2, GSR, OGDH, SDHB, PPP2R2A, HYPK, PRPSAP2, PFDN4, NCBP1, GLRX5, UQCRQ, FKBP2, PHPT1, SRSF11, RPL21, GALE, EMC1, SAMM50, CALU, ALDH9A1, MCM7, CDC37, HEXA, PLOD1, CYFIP1, CSTF3, COMT, SYMPK, VARS1, HNRNPLL, ACADS, SMU1, ABCE1, PSMB5, PSMB6, SF3A2, H3C1, DDI2, OSBPL8, EIF3M, POGLUT3, ACTR1A, NAE1, SNX1, FMNL1, ITGAV, HMGN5, SCFD1, CDC123, HDAC2, CTH, NNT, ABCC1, GOLGA4, DDX56, UBE2H, ME2, HADH, LTA4H, PRMT1, AKR1B1, TMEM123, PES1, FARSB, MTA1, HINT2, SPAG1, ISOC1, KIF20A, GRHPR, SYNE2, RPL27A, UBR4, GLO1, OGT, STX16, PYGL, SRM, STEAP4, KIF3A, PNPO, HACD3, CPT2, GYG1, SRSF10, TMEM14C, GLS, ACBD3, TTC1, COX6C, EIF5, SBNO1, VPS35, DARS2, FKBP1A, ARL3, ATP2B1, ATP1B1, C1orf122, GAGE2E, NPC1, LETM1, SPCS3, CKS1B, GHITM, HM13, SH3BGRL3, MTX1, ABCB7, AAAS, ITPR3, WDR43, FLII, MICU2, LRRC1, ARFIP2, PRKCI, SLC1A5, PSMD8, UBR5, RMDN3, DERL2, WDR1, DHX16 | AATF, ABL1, AES, AHR, AIP, AIRE, ANKRD1, APBB1, APC, APEX1, AR, ARID1A, ARID1B, ARID3A, ARNT, ARNTL, ARNTL2, ARX, ASCL1, ASH1L, ASXL1, ATF1, ATF2, ATF3, ATF4, ATF5, ATF6, ATF7, ATM, ATOH1, ATRX, BACH1, BACH2, BARD1, BARX2, BATF, BCL11A, BCL3, BCL6, BDP1, BHLHE41, BIN1, BMI1, BPTF, BRCA1, BRCA2, BRD7, BRIP1, BRPF1, BTAF1, BTF3, BTG2, CBFA2T3, CBFB, CBX7, CBX8, CDC5L, CDCA7L, CDX1, CDX2, CEBPA, CEBPB, CEBPD, CEBPE, CEBPG, CEBPZ, CHD4, CHD8, CIITA, CITED2, CLOCK, CNBP, CNOT7, CNOT8, COPS5, CRABP2, CREB1, CREB3, CREB3L1, CREB3L4, CREB5, CREBBP, CREG1, CREM, CRTC1, CRX, CTBP1, CTCF, CTCFL, CTNNB1, CTNNBIP1, CUX1, DACH1, DAXX, DBP, DDB1, DDB2, DDIT3, DDX5, DEAF1, DEDD, DEK, DENND4A, DLX3, DLX4, DLX5, DMAP1, DNMT1, DNMT3A, DNMT3L, DR1, DRAP1, DUX4, E2F1, E2F2, E2F3, E2F4, E2F5, E2F6, E2F7, E2F8, E4F1, EAF1, EAPP, EBF1, EBF3, ECD, EED, EGF, EGR1, EGR2, EGR3, EHF, EHMT2, EIF2AK2, ELF1, ELF2, ELF3, ELF4, ELK1, ELK3, ELK4, ELL, EN1, ENO1, EOMES, EP300, EPAS1, ERCC2, ERF, ERG, ESR1, ESR2, ESRRA, ESRRB, ESRRG, ETS1, ETS2, ETV1, ETV3, ETV4, ETV5, ETV6, ETV7, EVX1, EWSR1, EZH2, FHL2, FIGLA, FLI1, FOS, FOSB, FOSL1, FOSL2, FOXA1, FOXA2, FOXA3, FOXC1, FOXC2, FOXD3, FOXE1, FOXF1, FOXF2, FOXG1, FOXH1, FOXI1, FOXJ1, FOXK2, FOXL1, FOXL2, FOXM1, FOXN1, FOXN4, FOXO1, FOXO3, FOXO4, FOXP1, FOXP2, FOXP3, FOXQ1, FUBP1, FUBP3, FUS, GABPA, GABPB1, GABPB2, GATA1, GATA2, GATA3, GATA4, GATA5, GATA6, GBX2, GCM1, GCM2, GFI1, GFI1B, GLI1, GLI2, GLI3, GLIS3, GRHL1, GSC, GTF2A1, GTF2B, GTF2F1, GTF2I, GTF3A, GZF1, HAND2, HAX1, HBP1, HCFC1, HDAC1, HDAC10, HDAC11, HDAC2, HDAC3, HDAC4, HDAC5, HDAC7, HDAC9, HDGF, HES1, HES6, HEXIM1, HEY1, HEY2, HEYL, HHEX, HIC1, HIF1A, HIF3A, HINFP, HIPK2, HIRA, HIVEP2, HLF, HLTF, HLX, HMG20B, HMGA1, HMGA2, HMGB2, HMGN1, HNF1A, HNF1B, HNF4A, HNF4G, HNRNPD, HNRNPR, HOPX, HOXA1, HOXA10, HOXA11, HOXA4, HOXA5, HOXA7, HOXA9, HOXB1, HOXB13, HOXB4, HOXB7, HOXC10, HOXC11, HOXC13, HOXC6, HOXC8, HOXC9, HOXD1, HOXD13, HOXD3, HOXD9, HR, HSF1, HSF2, HSF4, HTATIP2, ID1, ID2, ID3, ID4, IFI16, IKBKB, IKZF1, ILF2, ILF3, ING1, ING2, ING4, IRF1, IRF2, IRF3, IRF4, IRF5, IRF6, IRF7, IRF8, IRF9, ISL1, JARID2, JDP2, JUN, JUNB, JUND, KAT2B, KAT5, KAT6A, KCNIP3, KDM2A, KDM4B, KDM4C, KHDRBS1, KHSRP, KLF1, KLF10, KLF11, KLF12, KLF13, KLF14, KLF15, KLF16, KLF2, KLF3, KLF4, KLF5, KLF6, KLF7, KLF8, KLF9, L3MBTL1, LCOR, LDB1, LEF1, LHX2, LHX3, LHX4, LIMD1, LIN28A, LMO2, LMO3, LMO4, LMX1B, LRF, LRRFIP1, LYL1, MAF, MAFA, MAFB, MAFF, MAFG, MAFK, MAL, MAML1, MAX, MAZ, MBD1, MBD2, MCM2, MCM5, MDM2, MDM4, MECP2, MED1, MED15, MED23, MEF2A, MEF2B, MEF2C, MEF2D, MEIS1, MEIS2, MEN1, MITF, MKL1, MLL2, MLL3, MLL4, MLLT10, MLLT3, MLX, MLXIPL, MSC, MSX1, MSX2, MTA1, MTA2, MTA3, MTF1, MXD1, MXD4, MZF1, MXI1, MYB, MYBBP1A, MYBL1, MYBL2, MYC, MYCN, MYF6, MYOCD, MYOD1, MYOG, NAB1, NAB2, NANOG, NCOA1, NCOA2, NCOA3, NCOA4, NCOA6, NCOR1, NCOR2, NEAT1, NELFB, NELFCD, NEUROD1, NEUROG3, NF1, NFAT5, NFATC1, NFATC2, NFATC3, NFE2, NFE2L1, NFE2L2, NFIA, NFIB, NFIC, NFIL3, NFIX, NFKB1, NFKB2, NFKBIA, NFKBIB, NFKBIZ, NFRKB, NFYA, NFYB, NFYC, NHLH2, NKX2-1, NKX2-2, NKX2-3, NKX2-5, NKX3-1, NONO, NOTCH3, NPAS2, NPAS3, NPM1, NR0B1, NR0B2, NR1D1, NR1H2, NR1H3, NR1H4, NR1I2, NR1I3, NR2C2, NR2E3, NR2F1, NR2F2, NR2F6, NR3C1, NR3C2, NR4A1, NR4A2, NR4A3, NR5A1, NR5A2, NRF1, NRIP1, NRL, NUPR1, OLIG2, ONECUT1, ONECUT2, OTX1, OTX2, PA2G4, PARP1, PAWR, PAX1, PAX2, PAX3, PAX4, PAX5, PAX6, PAX8, PBX1, PBX2, PCBD1, PCGF2, PDCD11, PDX1, PER1, PER2, PGR, PHB2, PHF10, PHF8, PHOX2A, PHOX2B, PIAS1, PIAS2, PIAS3, PIAS4, PIR, PITX1, PITX2, PITX3, PKNOX1, PLAG1, PLAGL1, PLAGL2, PML, POLR1A, POU1F1, POU2AF1, POU2F1, POU2F2, POU2F3, POU3F1, POU3F2, POU3F4, POU4F1, POU4F2, POU5F1, PPARA, PPARD, PPARG, PPARGC1A, PRDM1, PRDM14, PRDM2, PREB, PROX1, PTF1A, PTMA, PTTG1, PURA, RAD51, RARA, RARB, RARG, RB1, RB1CC1, RBBP7, RBL1, RBL2, RBMX, RBPJ, REL, RELA, RELB, REST, RFWD2, RFX1, RFX2, RFX3, RFX5, RFXANK, RFXAP, RNF14, RORA, RORC, RREB1, RUNX1, RUNX1T1, RUNX2, RUNX3, RUVBL1, RXRA, SALL3, SALL4, SALL4A, SATB1, SATB2, SCAND1, SCD5, SEC14L2, SERTAD1, SETBP1, SF1, SFPQ, SHOX, SHOX2, SIM2, SIN3A, SIRT1, SIRT2, SIRT3, SIX1, SKI, SKIL, SLA2, SLC2A4RG, SMAD1, SMAD2, SMAD3, SMAD4, SMAD7, SMARCA1, SMARCA4, SMARCB1, SMG6, SMURF2, SNAI1, SNAI2, SND1, SNIP1, SNW1, SOX10, SOX11, SOX17, SOX2, SOX4, SOX5, SOX6, SOX9, SP1, SP100, SP2, SP3, SP4, SP7, SPDEF, SPEN, SPI1, SPIB, SPIC, SRCAP, SREBF1, SREBF2, SRF, SRSF1, SRY, SSB, SSX2, STAT1, STAT2, STAT3, STAT4, STAT5, STAT5A, STAT5B, STAT6, SUGP1, SUPT3H, SUZ12, TAF1, TAF4, TAF5, TAL1, TBC1D22A, TBL1X, TBP, TBPL1, TBR1, TBX2, TBX20, TBX21, TBX3, TBX5, TCF12, TCF19, TCF3, TCF4, TCF7, TCF7L2, TCFL5, TEAD1, TEAD4, TEF, TFAP2A, TFAP2B, TFAP2C, TFAP4, TFCP2, TFCP2L1, TFDP1, TFDP3, TFEB, TFPT, TGIF1, THRA, THRB, TIAL1, TLE3, TLX1, TNFAIP3, TOB1, TOP2B, TP53, TP53BP1, TP63, TP65, TP73, TRAF6, TRERF1, TRIB3, TRIM16, TRIM22, TRIM25, TRIM28, TRIP6, TRPS1, TRRAP, TSC22D1, TSC22D3, TSG101, TTF2, TWIST1, TWIST2, UBP1, UBTF, UHRF1, UPF1, UPF2, URI1, USF1, USF2, UTF1, VDR, VEZF1, VHL, WDR5, WHSC1, WT1, WWP1, WWTR1, XBP1, XPC, XRCC5, XRCC6, YBX1, YEATS4, YY1, ZBTB14, ZBTB16, ZBTB17, ZBTB2, ZBTB5, ZBTB7A, ZEB1, ZEB2, ZFHX3, ZFP36, ZFP36L1, ZHX2, ZIC1, ZIC2, ZIC3, ZMYND11, ZNF143, ZNF148, ZNF160, ZNF175, ZNF202, ZNF217, ZNF224, ZNF239, ZNF24, ZNF267, ZNF300, ZNF335, ZNF350, ZNF382, ZNF383, ZNF410, ZNF423, ZNF444, ZNF76, ZNRD1, | RPS11, ERAL1, DDX27, DEK, PSMA6, TRIM56, TRIM71, UPF2, FARS2, FDXACB1, ALKBH8, ZNF579, POLR2B, WDR12, RPF1, MRPS34, UBTF, CDC5L, DAZAP1, MSI1, MSI2, TARDBP, TRIT1, MRPL36, KPNB1, ZNFX1, SNRNP27, RBMS2, RBMS1, RBMS3, LARS2, KIAA0020, RBFOX1, RBFOX2, RBFOX3, CWF19L1, CWF19L2, ZC3H15, LONP1, DBR1, RRP15, L1TD1, NMD3, SRSF10, SRSF12, SRSF2, SRSF8, RSL1D1, DAP3, NARS2, RBM25, POLR2E, TOE1, TSFM, TPR, STAU1, STAU2, TRMT1L, METTL3, REXO2, MRPL52, WDR3, CNOT11, YTHDC1, METTL2A, METTL2B, ZNF326, AKAP8, AKAP8L, RPS14, ELAVL1, ELAVL2, ELAVL3, ELAVL4, PNN, DDX41, TNPO2, TNPO1, PUSL1, RBM43, ZGPAT, SCAF11, USP39, RPL38, NARS, RPS16, CLASRP, RPS9, TOP3B, NCBP2L, NCBP2, SMG9, FTSJ2, DDX56, RBM28, UTP11L, MRPS18B, RPL14, RPL37, GEMIN5, MKRN1, MKRN2, MKRN3, NOA1, PINX1, HRSP12, TDRD9, DHX30, DHX29, DHX57, YTHDC2, DHX36, DHX9, EIF3G, NOL10, UTP15, GATC, TCOF1, NONO, PSPC1, SFPQ, NUDT21, MRPL19, URM1, MRPL53, RBM48, REPIN1, ZC3H12B, ZC3H12C, ZC3H12D, ZC3H12A, TRMT5, TRMT61A, PABPN1L, PABPN1, LSG1, GNL1, EEF1G, RBM33, RPP25, RPP25L, RPP38, GARS, DRG2, TRMU, GNL2, CPSF2, CD3EAP, HDLBP, RDM1, BYSL, GEMIN8, XPO5, ZNF622, DZIP1, DZIP1L, GSPT1, HBS1L, GSPT2, EIF2D, METTL1, RNASEH1, ZNHIT6, DARS2, SNRPD2, DICER1, DYNLL1, ZNF638, SAMD4B, SAMD4A, RTF1, CNOT4, TARBP2, PRKRA, GLE1, EXOSC2, SSU72, GTPBP10, RPL10A, HENMT1, THOC2, ENDOG, EXOG, ASH1L, TRMT10C, TRMT10A, TRMT10B, MRPL33, SSB, LARP7, MRPL24, RNASEK, PELO, QARS, IPO4, CAPRIN1, CAPRIN2, PNLDC1, PARN, TIAL1, TIA1, CDC40, ZNF598, RCL1, RTCA, MRPL38, ADAT2, BOP1, U2SURP, DUS3L, CSTF3, SRSF11, SREK1, POLR1E, EXOSC7, EXOSC8, EXOSC9, MRPL12, RPS28, TEFM, RPL12, ZC3H3, EIF6, NELFE, EIF2B3, APOBEC2, APOBEC3H, APOBEC3F, APOBEC3G, APOBEC1, ZC3H7A, ZC3H7B, NOP9, PQBP1, PATL2, PATL1, EDC3, WBP4, GLTSCR2, RPS18, ASCC1, EIF5A, EIF5A2, EIF5AL1, MRPL47, BOLL, DAZL, DAZ1, DAZ2, DAZ3, DAZ4, INTS1, RPLP1, MRPS11, WDR43, TRMT112, PPP1R8, FBXO17, MTHFSD, SON, SPATS2L, SPATS2, MAEL, RRP1B, RRP1, ZC3HAV1, PARP12, TIPARP, MRPS17, U2AF2, UHMK1, NUP153, CSDE1, POLR2L, NKRF, MRPL48, XRCC6, SUPT5H, TSR3, XRN2, RSRC1, TDRD5, POLR2J, POLR2J2, POLR2J3, SAFB2, SAFB, SLTM, BRIX1, POLR2I, PWP2, ELAC2, ZCCHC14, ZCCHC2, LARS, MRPL28, CNOT1, DDX18, TSEN15, USP10, SMNDC1, THUMPD2, THUMPD3, SND1, LSM4, EIF3M, MRPL34, SRP72, SUGP1, SUGP2, RPS3, RPS27A, CCAR2, CCAR1, DDX49, MRPL4, SFSWAP, MRPS12, EIF1, EIF1B, CLP1, ZMAT5, SF3B3, PLD6, QTRTD1, QTRT1, PIH1D1, SWT1, EXOSC4, EXOSC6, PTRH1, INTS8, EXOSC10, CASC3, GTF3A, MRPL54, GTPBP3, UBA1, LSM12, HINT3, CCDC59, POLDIP3, RPL32, AZGP1, EMG1, C17orf85, N4BP1, NYNRIN, KHNYN, NOP2, NSUN4, NSUN3, NSUN6, PAN2, ZCCHC9, RRP9, TNRC6A, TNRC6B, TNRC6C, RP9, MRPL15, NGDN, LSM10, PUS1, MAK16, RPS13, SNRNP200, PRDX1, TSN, NUFIP2, RBM19, TDRKH, NOP58, RBM6, RBM10, RBM5, RBM44, OASL, OAS1, OAS2, OAS3, SF3A3, NOC3L, MYEF2, HNRNPM, C11orf68, MRPL10, RNASE10, CALR, CANX, CALR3, RPP14, RPS8, PDCD7, RPS19BP1, TRMT6, RPL4, PRPF3, RARS, RARS2, APOBEC4, ZCCHC24, RPS21, DDX24, RTCB, MBNL2, MBNL3, MBNL1, ALYREF, LSMD1, PUS7, PUS7L, MRPS24, RBBP6, RPL19, NOL3, RPS27, RPS27L, PTRF, MRPS18A, SLBP, EARS2, SMAD1, SMAD2, SMAD5, SMAD9, SMAD3, SMAD4, SMAD7, SMAD6, PRPF39, SRP68, ADARB2, ADAD1, ADAD2, ADAT1, ADARB1, ADAR, CLK3, CLK4, PRPF4B, CLK1, CLK2, LSM3, FAM46A, CRYZ, KIN, RPS2, DUS4L, EIF4A1, EIF4A2, EIF4A3, IGF2BP1, IGF2BP2, IGF2BP3, UBAP2, UBAP2L, SNRPE, EIF2B5, CRNKL1, POLRMT, METTL10, MRPL41, MRPS7, NSUN5, NSUN7, MTG1, MRPS28, TDRD10, RPUSD1, NHP2, XAB2, FAU, RRP36, GRWD1, ENDOV, VARSL, MRRF, MTFMT, C12orf65, MRTO4, RBM26, RBM27, DIS3, DIS3L, DIS3L2, C1QBP, SUB1, MRPS33, PRPF6, SMG8, PSTK, GAPDH, TNPO3, IPO13, MOV10, MOV10L1, PARP1, MRPS35, GPKOW, SETD1A, SETD1B, SF3A2, AFF1, AFF3, AFF4, AFF2, PARK7, TRNAU1AP, SRBD1, EIF3D, NOP10, WARS, MATR3, SNRPD1, SNRPC, FXR1, FXR2, FMR1, MEPCE, KAT8, CIRBP, RBM3, CNOT7, CNOT8, A1CF, RBM46, RBM47, DND1, HNRNPR, SYNCRIP, ABCE1, GPATCH4, PAIP1, RBM20, GNL3, GNL3L, FAM120A, FAM120B, FAM120C, KHDC1, KHDC1L, RPL10L, RPL10, EIF2S1, MRPL20, EPRS, DHX58, DDX58, IFIH1, CDK5RAP1, HABP4, SERBP1, METTL5, ZCCHC7, WDR36, CCDC86, C2orf15, MRPL30, RPS26, TEX13A, BARD1, XPO4, RUVBL1, IMP4, DDX52, THOC1, TRPT1, NHP2L1, EIF2B2, RNF113B, RNF113A, INTS10, LENG9, RPS29, NOP16, U2AF1, U2AF1L4, RANBP6, IPO5, THOC7, DNMT3B, GFM1, RNASE9, PLRG1, REXO1, ZC3HAV1L, LSM14A, LSM14B, LUZP4, TDRD6, TDRD15, MRPS22, EEF1B2, EEF1D, SIDT1, SIDT2, TRMT44, GPATCH8, WDR4, SLIRP, ATXN1, ATXN1L, YTHDF1, YTHDF2, YTHDF3, LRPPRC, TFIP11, ILF2, STRBP, ILF3, TCERG1, NXF5, NXF2B, NXF2, NXF1, NXF3, ZRSR1, ZRSR2, MRPL35, RRS1, PRKDC, NOC2L, MTIF3, NOL11, EXOSC3, PNPT1, TSNAX, RIOK1, RIOK3, PIWIL1, PIWIL2, PIWIL3, PIWIL4, PEG10, ZCCHC5, POP7, FASTKD3, TBRG4, FASTK, TRMT13, IREB2, ACO1, ZCCHC3, SNUPN, PTRHD1, HARS, HARS2, HNRNPK, PCBP1, PCBP2, PCBP3, PCBP4, AQR, ICT1, UNK, UNKL, CEBPZ, MRPS9, THG1L, ZCCHC13, CNBP, SRRT, CD2BP2, SNRPG, APTX, RPL17, BMS1, SARS, SARS2, PAIP2, PAIP2B, GEMIN4, PNRC2, FASTKD2, GEMIN6, DDX20, DDX39A, DDX39B, DHX37, ZNF346, MRPL50, RNASE1, RNASE2, RNASE3, RNASE4, RNASE6, RNASE7, RNASE8, ANG, DYNC1H1, FTSJ3, SF3B2, CTU2, XPOT, RBMXL1, RBMXL2, RBMXL3, RBMY1A1, RBMY1B, RBMY1D, RBMY1E, RBMY1F, RBMY1J, RBMX, PPP1R10, PRR3, DKC1, MRPL17, TRA2A, TRA2B, RPL26L1, RPL26, AUH, EIF3C, EIF3CL, SCAF8, SCAF4, EEF2K, RPL6, MRPL11, G3BP1, G3BP2, MRPS26, SF3B5, DHX34, UTP23, SNRNP48, PRPF38B, HEXIM1, HEXIM2, SRRM2, SRRM3, PIH1D3, DHX40, DHX38, TRMT61B, CSDC2, CARHSP1, POP4, LARP6, RNPC3, RPL30, RUVBL2, DRG1, TRIM25, RQCD1, MRPS18C, MRPL45, RPLP2, SRP19, RPS24, AKAP17A, NXT2, NXT1, MRPL9, RPL27, RPL24, CNOT10, ABT1, DDX25, DDX19A, DDX19B, MRPS30, SMG1, EXOSC5, AIMP2, BZW2, BZW1, PARS2, INTS12, EIF3H, SKIV2L, RC3H2, RC3H1, ZFP36, ZFP36L1, ZFP36L2, ANKHD1, ANKRD17, HELZ2, UPF1, DROSHA, MRPL37, PUS10, NUDT16, NUDT16L1, TRMT11, PET112, NUFIP1, PURA, PURB, PURG, ZRANB2, THOC5, TSEN54, SRP9, PIN4, ZC3H14, CNOT2, TARBP1, AGO1, AGO2, AGO3, AGO4, LARP4B, LARP4, SNW1, EZH2, EIF3B, RPP30, ZMAT3, PTGES3, KARS, RAN, SNRPA, SNRPB2, HEATR1, PTGES3L-AARSD1, AARSD1, CPSF3, CPSF3L, CWC25, RPS5, POLR2D, C9orf114, MRPL44, ETF1, GAR1, MVP, RPF2, ZC3H18, INTS5, INTS3, LSM6, SNRPF, TUT1, MTPAP, EIF2AK1, EIF2AK2, EIF2AK3, EIF2AK4, MRPL22, PES1, LSM7, PAPD4, ZCCHC11, ZCCHC6, KHDRBS1, KHDRBS2, KHDRBS3, QKI, SF1, LRRC47, ISY1, SRRM1, IPO9, MTRF1, MTRF1L, WIBG, PPAN, FUBP3, FUBP1, KHSRP, CHTOP, PTCD2, POLR2A, MRPL43, DDX55, RPS15, CPSF4, CPSF4L, CSTF2, CSTF2T, RAVER2, RAVER1, EIF1AX, EIF1AY, DALRD3, NSUN2, URB2, PHAX, PPARGC1A, PPARGC1B, PPRC1, PHF5A, MCTS1, RRP7A, DGCR8, ZC3H13, UPF3A, UPF3B, RBM24, RBM38, THOC3, IGHMBP2, PAPD5, PAPD7, GFM2, PHRF1, VARS, VARS2, MRPL1, INTS7, ATXN2L, ATXN2, PPIL4, PPIL3, PPWD1, CWC27, PPIH, NUTF2, MRPL18, LSM11, AARS2, AARS, MIF4GD, CTIF, C9orf129, LUC7L2, LUC7L3, LUC7L, DUS2, RNF17, TDRD1, MRPS10, FAM103A1, ENOX1, ENOX2, RBPMS, RBPMS2, NIP7, RPL3, RPL3L, RNASE11, RNASE12, RPLP0, RBM34, CMTR2, ENDOU, PUM1, PUM2, TYW5, TYW3, TRMT1, RAE1, SLU7, EIF2B4, EIF5B, GPATCH1, RPL36, EEF1A1, EEF1A2, RRP8, UBA52, R3HDM1, R3HDM2, PA2G4, AAR2, ERI1, ERI2, ERI3, NIFK, METTL14, MRPL32, CPSF6, CPSF7, RPL7A, WDR46, SRP14, TFAM, CMSS1, XPO6, EXOSC1, ZNF106, UTP14A, UTP14C, TRUB1, TRUB2, PRIM1, PTCD3, HNRNPA0, HNRNPA1, HNRNPA1L2, HNRNPA2B1, HNRNPA3, HNRNPAB, HNRNPD, HNRNPDL, RANBP2, RPL7, RPL7L1, RNASE13, RPS6, MRPL3, DDX28, DDX47, RNASEH2C, TFB2M, TEP1, NOC4L, CPSF1, DDX1, DDX3X, DDX3Y, DDX4, NOP56, RPS20, MPHOSPH10, MRM1, DXO, DENR, RPL21, RPS10, NAT10, UTP18, NOL12, DDX31, ABCF1, DNMT1, SNRPA1, API5, SHQ1, AKAP1, FTO, PUF60, SAMHD1, RBM45, FAM98B, FAM98A, FAM98C, PCF11, EIF5, ZC3HC1, HNRNPC, HNRNPCL1, RALY, RALYL, SEPSECS, NPM1, NPM3, NPM2, NUPL2, MRPS14, SF3A1, EIF3A, FASTKD5, TDRD3, SNRPB, SNRPN, YRDC, POLR2K, ERN2, ERN1, RPL5, RPS17, RPS17L, DARS, IPO7, IPO8, SMN1, SMN2, GTPBP4, MRPL13, MRPL46, LSM5, EIF4G3, EIF4G1, EIF4G2, GTF2F1, RPS15A, PIH1D2, TTF2, PUS3, RBM7, RBM11, TSEN2, RBM42, UTP3, HNRNPL, HNRNPLL, PTBP1, PTBP2, PTBP3, SYMPK, POLR2F, TSR2, KIAA0391, POLR2G, WDR5, SNRNP40, WDR83, ZFC3H1, RPL15, SNRNP35, SNRNP70, MRPL39, TLR7, TLR8, TLR3, NAA38, PRPF31, MRPL55, TRMT2A, TRMT2B, RPS3A, BRCA1, RIOK2, DHX16, DHX33, DHX35, DHX32, DQX1, DHX15, DHX8, MRPS5, SAP18, TUFM, EEFSEC, TDRD12, SKIV2L2, TRDMT1, ZNF473, FCF1, URB1, CARS, CARS2, CELF1, CELF2, CELF3, CELF4, CELF5, CELF6, XPO1, DDX17, DDX46, DDX5, DDX42, DDX43, DDX53, FIP1L1, MRPL14, TXNL4A, TXNL4B, RBM39, RBM23, CPEB1, CPEB2, CPEB3, CPEB4, INTS9, CACTIN, MPHOSPH6, RPL34, OBFC1, RPS12, PRPF8, SECISBP2L, SECISBP2, RPUSD3, RPUSD4, DDX6, RPL28, ELAC1, NOM1, TROVE2, EEF1E1, TARSL2, TARS, TARS2, IARS, RPL18A, ESF1, EEF2, EFTUD2, DDX60, DDX60L, MRPL2, SLC4A1AP, MAZ, EIF2S3L, EIF2S3, AIMP1, APEX1, EIF3J, PABPC3, PABPC1L, PABPC1L2A, PABPC1L2B, PABPC4L, PABPC5, PABPC4, PABPC1, MEX3A, MEX3B, MEX3C, MEX3D, GEMIN7, MRPS23, PSIP1, SRP54, FARSB, SPEN, RBM15B, RBM15, DNAAF2, SF3B1, RNASEH2B, FARSA, NOB1, EIF2A, RNASEL, TRNT1, EIF4ENIF1, MECP2, RBM22, CMTR1, RPL39, RPL39L, RPSA, PARP4, CSTF1, NCBP1, SNRNP25, DNAJC21, CWC22, NFX1, MRPS31, SMG5, SART1, GEMIN2, NR0B1, DCP2, TAF15, EWSR1, FUS, EDC4, FBLL1, FBL, MRPS27, ALKBH1, ZCCHC17, MRPL49, RPL18, RPS4X, RPS4Y1, RPS4Y2, NOP14, KRR1, NCL, PRPF18, C1D, ADAT3, UTP20, SNIP1, GUF1, GCFC2, UTP6, SRSF1, SRSF3, SRSF4, SRSF5, SRSF6, SRSF7, SRSF9, USB1, NOL6, PPIE, RRBP1, SUZ12, TERT, MSL3, BICC1, MRPS16, RBMX2, MRPL40, ISG20, ISG20L2, AEN, REXO4, TGS1, ZC3H4, ZC3H6, ZC3H8, RPL27A, RPS19, SYF2, TFB1M, DIMT1, TSEN34, DCAF13, RPL9, ARHGEF28, AC004381.6, EIF4H, EIF4B, DDX54, MRPS36, PRPF40A, PRPF40B, EIF3L, NOL8, MTO1, PSMA1, HTATSF1, FTSJ1, LAS1L, POP1, DZIP3, DGCR14, CDK9, PAN3, RPL29, DCPS, MRP63, MRPL27, POLR2H, ZNF239, XRN1, ZFR, ZFR2, AGFG1, NSA2, DDX10, TBL3, INTS2, YBX1, YBX2, YBX3, SURF6, THOC6, EIF3I, STRAP, RPL13, RRP12, MRPS2, ZCCHC8, EFTUD1, RPL35, NSRP1, RPUSD2, BCDIN3D, THUMPD1, TRIM21, RANBP17, XPO7, TRMT12, ACIN1, ZNF385A, RPL23, EED, DNTTIP2, SBDS, DDX51, RBM18, IPO11, RPL22, RPL22L1, LCMT2, ZC3H11A, FYTTD1, RNPS1, RPS23, RNASEH2A, FRG1B, FRG1, NOL7, PRPF38A, RBM41, RBM12, RBM12B, ESRP1, ESRP2, HNRNPF, HNRNPH1, HNRNPH2, HNRNPH3, GRSF1, RNH1, NOL9, SRFBP1, ARL6IP4, RPP40, ALKBH5, SMG7, PDE12, CCRN4L, ANGEL1, ANGEL2, CNOT6, CNOT6L, PDCD11, RPL35A, MRPL21, SMG6, PRPF19, PRPF4, INTS4, SRPK2, SUPV3L1, RBM17, NAF1, YARS, LRRFIP1, LRRFIP2, EIF4E2, EIF4E1B, EIF4E3, EIF4E, BUD13, CTU1, GTPBP1, GTPBP2, MTERFD2, SRPR, DDX50, DDX21, ZCRB1, RPL13A, SRA1, PAPOLB, PAPOLG, PAPOLA, RPL41, ZNF768, SCAF1, MRPS15, RNASET2, EIF2B1, RPL36A, RPL36AL, DDX26B, INTS6, RPS7, RRNAD1, MRPS21, SETD7, RBM8A, MAGOH, MAGOHB, RPL37A, NOLC1, IARS2, LSM1, SUPT4H1, EXO1, DCP1B, DCP1A, DNAJC17, RNGTT, DUSP11, CHERP, WDR61, NANOS1, NANOS2, NANOS3, EBNA1BP2, HELZ, RNMTL1, PDCD4, PTRH2, SART3, ZC3H10, LSM2, MRPS25, RPP21, ZMAT2, IFIT1, IFIT2, IFIT5, IFIT1B, IFIT3, NOVA1, NOVA2, SNRPD3, YARS2, PTCD1, TSR1, MRPS6, TAF9, RPL11, DDX23, DDX59, CCNT2, CCNT1, RPL8, SARNP, IMP3, SRRM4, CNP, LIN28A, LIN28B, EIF3K, SF3B14, EIF2S2, RNMT, MRPL42, THRAP3, BCLAF1, CXorf23, PNO1, RBM4, RBM4B, RBM14, R3HCC1, R3HCC1L, KIAA0430, POP5, MRPL51, MARS, TYW1, MRPL16, DUS1L, QRSL1, WARS2, WRAP53, RSL24D1, SF3B4, TDRD7, LARP1, LARP1B, RPL23A, BAZ2A, BAZ2B, EIF3E, CWC15, TOP1, RPL31, SUPT6H, TST, HNRNPU, HNRNPUL1, HNRNPUL2, RPS25, MRPL23, EIF1AD, SETX, MARS2, CNOT3, MTIF2, JAKMIP1, FASTKD1 | EED, SUZ12, UPF2, DNMT1, KHDRBS1, EZH2, SRSF1, KHSRP, HEXIM1 |

**Supplementary Table S6. Screening for circGSK3B targets by intersecting DEGs with biogrid targets or tftargets.**

| Differentially expressed genes (DEGs) | Biogrid | tftargets | DEGs intersect Biogrid | DEGs intersect tftargets | DEGs intersect (Biogrid OR tftargets) |
| --- | --- | --- | --- | --- | --- |
| GSK3B, CHAC1, AC112191.1, AC004069.1, AC027139.1, TMEM255B, OVCH1-AS1, TCP11L2, HSPA8, PSPH, CARS1, SESN2, CTH, STC2, GDF15, GPT2, AC135506.1, SLC1A4, PHGDH, AC107398.2, WDR86, MLXIPL, GARS1, ABCG1, BX539320.1, PSAT1, ADM2, LINC00662, AL354696.2, PTPN12, AL354872.1, NUPR1, VLDLR, SLC6A9, KIF21B, CAMK2N1, LINC01667, COX6B2, ANG, CHST6, AC026462.1, ULK1, BX640514.2, NWD1, AC006504.1, CEBPG, LURAP1L, H1-0, OSBPL6, CHST2, AC245100.3, ARHGAP25, CBS, CAPN11, CHMP4BP1, KCNJ18, ASNS, COL4A3, ALDH1L2, H4C14, BBC3, HOXB9, SLC7A11, HERPUD1, CLEC2D, CD163L1, DUSP8, EGR1, LINC00862, NEURL1B, CDKN2B, IGFL1P1, PHF21A, AL031985.3, IFRD1, DOP1B, CDKN1A, DAPK1, UHRF1BP1, PROX2, AK7, MTHFD2, GNG13, TSC22D3, AC144652.1, AP006545.1, SH3BP2, GPRC5B, PPARGC1A, VEGFA, AC096887.2, AC090015.1, JDP2, LINC01138, TRIB3, GRB10, RNF144A, AC090241.3, MARS1, DGCR10, CHMP1B2P, DDIT3, SNED1, GNAO1, CYBRD1, LZTS3, ID2-AS1, ATF4P3, AC103706.1, ERRFI1, RORB, KLF12, ATXN1, PCK2, FAM9B, CXCR4, TMEM86A, PGPEP1, AL109615.2, AL109615.3, RHOT1P1, GEM, NIBAN1, AC131009.2, AC100821.2, ARNT2, DGCR5, AC019080.3, LINC00526, AC013762.1, XBP1P1, FZD10, AC148477.2, AC020910.5, LMO4, AC108448.2, AP004609.3, PCDH1, HAGLR, H2AW, BMP6, AL354919.2, AC010536.3, KNDC1, MCF2L, AC010618.3, ATF3, N4BP3, AL627309.7, SCN4A, PNMA2, USP32P3, AL133406.2, IL20RB, AC127024.8, NDUFA4L2, KLF9, AC022126.1, AC103974.1, ERFL, ERFE, AL356234.3, ULBP1, P2RX1, SARS1, FZD10-AS1, AC061975.8, CASKIN1, LRFN1, ETV5, SLC7A2, H1-10, AC067852.5, NTN4, SHMT2, HOXB2, PADI1, AL135924.2, HRK, AC005089.1, EYS, TRAF1, AC091153.3, INHBE, SATB2, AL355312.6, KRT16, EOMES, SLC43A1, AC096669.1, AL031282.2, GABPB1-IT1, TPBG, H2AZ1-DT, KIAA0513, NDRG1, MLANA, TMEM154, BTBD19, AL354821.1, ABCA1, ZSCAN12P1, RARB, AC132872.1, AP000424.1, SLC6A16, DGKZP1, AL121827.2, AC114930.1, AL162311.3, RNF125, UNC5A, DLGAP1-AS2, AC090229.1, AL589787.1, AC104758.1, ADAM1B, AL050341.2, BBIP1P1, ENTPD8, AC148477.3, FOXP1, ARHGDIB, C1QTNF1, LINC01293, PLGLB2, YARS1, SNAP25, SLC2A3, LINC02846, CXCL8, IGFBP1, CARS1-AS1, HSD17B1P1, CLDN7, RNA5SP123, FHL1, ZFP69B, AC126696.3, AARS1, CCDC39, SLC2A1-AS1, LINC01962, TESK2, ARHGAP4, AP001178.1, SH3GL1P1, KCNT1, H3C7, AC105206.2, SDR16C5, H2BU1, TSPAN5, PCDHB15, MERTK, MAB21L3, PPIL6, AC107959.3, AC009962.1, DENND5B-AS1, UNC5CL, MIR194-2HG, NRTN, FBXL13, AC117500.3, LINC01881, MTMR3, LINC01273, H2BC9, RPL13AP20, CD96, LINC00520, AL138756.1, LINC01886, AL442067.3, PYY2, AC009093.1, ECM2, HOXD11, AC108727.1, LINC01622, ZDBF2, SLFN5, KLK14, AC024270.5, FBXO2, NPM1P35, EIF4HP1, AC092683.1, SERPINB9P1, AC131212.2, MISP3, CLGN, LINC01460, PIP5K1B, FBXO32, HYAL4, GRK3, ZNF132, S100A14, PIP5KL1, HEY1, LINC02733, AC018638.6, AP001626.1, SMC1B, ZC3H6, ZNF821, ID3, ERBB4, LINC01094, AL031777.1, CA13, AC139099.3, AC005332.4, MKKS, TMEM200B, CBFA2T3, SLC7A11-AS1, RNU7-40P, AC138696.2, AL606500.1, SMIM14, AC016394.4, MOCOS, LUCAT1, AC131159.1, AC009302.1, LARP6, TUBE1, ANKRD2, PICART1, AL356356.1, INPP4B, IGFL2-AS1, AC046134.2, ACTN3, MIR3189, SESN3, KCNH1-IT1, ZFHX4-AS1, AC036214.1, SELENOP, AC119396.1, AC023855.1, LINC02367, ZNF572, ATXN1-AS1, SNTB1, CYP51A1-AS1, RASGRP2, SNORA23, LINC01422, TTC24, DNAJB13, CA15P1, AC243964.3, AC068580.5, CYP1B1-AS1, CAPN8, IGSF22, PXK, SPX, SCGB2B2, AC027288.3, AC009113.1, AL663070.2, IL1R1, CYP39A1, AC127496.1, GALNT9, AL592295.4, AC018470.1, AC026336.3, OLAH, AC020916.2, AJ003147.3, AMDHD1, NECAB2, AC025181.2, HDHD2, KRT34, AL022724.3, STPG4, AC023043.2, AC104532.2, TUBB2B, AL158825.2, SHISAL1, AC244669.1, OSER1-DT, WFIKKN1, AC004840.1, HAL, FHOD3, AL139317.4, PLXDC2, BOC, USP50, RN7SL517P, FAM27C, OR2A9P, ZNF467, TWIST1, BX255925.4, SYCE1, AC073439.1, AL591926.3, UQCRBP1, LINC01979, AC078925.3, NCAN, CALML6, AL353588.1, AC121757.2, AC090192.2, SP8, KANK4, SLCO1C1, RPL11P3, ADAMTS10, CYS1, AL513365.1, TFF1, TSPAN18, AC012358.2, AC007785.1, WFDC1, AKR1B10, AC127035.1, AC046143.2, LURAP1L-AS1, AC137936.1, AC005020.1, PCK1, PTGER1, AC008537.2, SERTAD4, AL353662.2, KLF7, AC006059.5, AC093307.1, ERVMER34-1, AC135050.7, TTLL13P, AC093827.4, IQCH-AS1, AC007493.2, HAR1B, SLC16A11, AL034417.5, TMEM251, RHOT1P2, AC103923.1, PAPPA, GJA3, AC010201.2, AL021807.1, AL133297.2, RERG, INSRR, TACR2, TXNIP, AL021707.7, AC009137.2, PPFIA4, AL445471.1, AC011468.4, WFDC21P, WDR31, CCT6P3, AC019080.1, LINC02269, HNF1A, IL17REL, CFL1P5, ZBTB12BP, LINC02328, AC127024.5, AC016644.1, TPRG1, NTSR1, H2BC17, AC092687.3, SH2D6, AL354740.1, PRR34, HIF1A-AS3, AC106820.5, HOXD8, AC019080.4, TCAP, EEF1A1P12, SLC44A3-AS1, AL591623.1, PADI2, BEST1, AC022217.4, ZNF775, AP005061.2, MYZAP, OMG, AC108463.1, PCDHGA1, CR381653.2, AC027020.2, AC016026.2, CMTM7, AC009090.3, RORA, TMPRSS4, EDIL3-DT, AC022400.4, HSPD1P4, LINC00322, AC121757.1, OTUD7A, EIF4EBP3, GDPD1, PARD6G-AS1, AC010478.1, LINC02806, PRTN3, TOB1-AS1, LINC01227, AC244033.2, SLIT1, AC012557.1, AL360270.2, POU6F1, PSAPL1, AC009549.1, ATXN2-AS, AL353763.1, AL031432.4, ZMIZ1-AS1, MIR6797, AC116021.1, AL137003.1, SLC45A1, BMF, FXYD3, KLHDC1, SNORD14E, AC002480.1, ACTBP13, PLS3-AS1, PCDHB11, BARX1, AL139234.1, AP000919.3, AC020658.6, AC027682.7, AC068282.2, IL17D, PIWIL2, MIR210HG, CACNG6, AC005076.1, SLC47A1P2, AL845552.2, AP003071.1, VSTM5, AC127537.1, AC188617.1, SARNP, AC018695.6, APOBEC2, AC007610.6, KDM7A-DT, GTF2IRD2P1, CLDN9, MAN1C1, RNA5SP18, NPIPB6, BDKRB2, CRYZL2P, MASP1, PDCL3P4, AC034187.1, LINC02481, AL591845.1, GZMM, TH, TSPAN19, AP002851.1, AC068491.3, ABLIM2, CDH16, AC007383.1, SND1-IT1, RPL7AP66, LINC01869, AC092645.1, FOXP2, DEPTOR, AP000238.1, AP001981.2, FAM228B, AC000123.2, LDHAL6A, SAP30-DT, RPS15AP10, LPL, SUMO1P3, LINC00853, AC022506.2, AL353804.1, LINC00304, SLC34A3, RWDD4P1, THEM5, LRMDA, IPO7P2, WNT10A, AC139768.1, AC004466.1, ZNF890P, SPACA6P-AS, AC008267.3, AC104971.1, CDH3, F2, AC005326.1, NTN3, NLRP6, WNT4, NOG, CEP83-DT, AL135999.1, AC015983.2, STC1, AC016710.2, AL021392.1, SIGLEC1, ACTA1, KCNH2, LINC02678, LINC01559, AC034213.1, LINC02245, SH2D7, AC005330.1, AC020978.1, LRRC32, FAM27E3, AC025171.5, HS6ST3, GHET1, RNU6-431P, AL161725.1, AC007688.2, RASGRF2, WWTR1-AS1, IL31RA, BX322635.1, ADGRD1, AC135721.1, DNAJC9-AS1, TRPV2, ZNF425, CCDC144CP, SERPINB4, SSTR5-AS1, ARHGAP23P1, MESP2, AL596223.2, AC026992.2, PDE6C, CEBPB-AS1, INHBA, RGMA, CORO7, FES, AL513550.1, SYT15, AP006248.4, MAMSTR, AC092849.2, CD70, AP002992.1, SIGLEC15, BEST3, ARL17A, AC083964.2, IL20RB-AS1, PABPC3, PTRH1, LINC02872, AC010615.1, AC026368.1, SGPP2, NDST3, IL20, GBP4, AL358473.1, CKS1BP3, AL157394.3, LINC01134, OMD, HOXA2, LINC02466, AC018926.2, AL117336.1, AC083805.2, AL117336.2, AP001350.2, SETSIP, ERICD, CA9, AL596094.1, ARHGEF2-AS1, SLC25A34, AL021154.1, RSPO3, PRSS8, AL713899.1, OVOL2, ARG2, AC046185.3, GPANK1, AC113208.6, AC112128.1, IFITM10, PRKCG, FOXO6-AS1, AL121845.4, AC138819.1, AC027373.1, LINC02757, LNCSRLR, CPEB1, AC105001.1, FBXW12, AL445072.1, AC092811.1, AC016773.2, FGF11, AC092301.1, AL513485.1, AC131009.4, DAW1, LINC02019, AP000812.1, AC004678.1, LINC02793, PPM1J, AC005546.1, AC005086.2, PAUPAR, AC092045.1, CPAMD8, AP002761.3, AP006222.1, CD7, Z98745.2, CRHR2, AL359643.2, LINC01970, EPHA6, FO681492.1, AL022393.1, ZNF687-AS1, HSPA8P5, TNFSF4, AL591686.2, IL24, AL359715.1, RASGEF1C, AL355596.1, AL031668.1, C14orf132, AC103758.1, LRP1B, AC069544.1, CHRNB4, AC090948.4, LINC02521, RAD21-AS1, MFF-DT, SPARC, AC069234.1, AL121829.2, CYP2F2P, AC091390.4, MYT1L, DDX53, AL365217.1, AC092119.2, SAMD14, BACH1-AS1, RAB11FIP4, LINC00926, AL360181.1, AC023043.3, LINC02175, ETV5-AS1, OVCH1, GALNTL6, AC090541.1, LINC00482, LINC00638, AL136164.3, RPL5P1, LINC01943, TMEM63C, AL031846.2, ZNF236-DT, CLDN10, AL078581.4, AL391095.4, AC009948.4, CCR3, SNORA38B, AC068790.4, PPP1R36, AC087190.2, AC073575.4, AC008481.1, PRDM6, AC009055.2, AC113208.4, UVRAG-DT, CXCL2, AC025370.2, AC144836.1, AC068669.1, AC090510.4, C3AR1, AC004877.2, SLC34A1, TSPYL6, GCGR, AC005696.4, AC004241.5, SMIM32, AL136038.7, CPQ, SLC23A3, AC105460.2, AL121938.1, NQO2-AS1, AC114341.1, LMO7-AS1, SLC12A3, AC118553.2, RPS6P16, MIR4324, AC105206.1, AC022432.1, LIPE-AS1, AC007279.2, RNU6-1262P, RPS26P6, AC024575.2, AL358933.1, VLDLR-AS1, C22orf23, AC092670.1, GFI1, LINC00705, AL136295.6, TOB2P1, KY, AC114489.1, SLFN13, DCDC2B, AC004967.1, SCAMP5, AL035603.1, AC105020.5, Z73965.1, BHMG1, GPR68, FAM167B, PPM1AP1, ERICH3, GPR62, LINC01796, AC093330.3, AC126124.2, AC011676.2, AC020765.4, COL8A2, AC078850.1, LINC02783, RN7SL541P, AC009041.1, ARHGAP8, AL162426.1, MIR7976, PRKG2, BTF3P7, PIGZ, AC079949.5, AC016747.2, AC119396.2, AC254633.1, AL359922.3, UBE2SP2, LCMT1-AS1, GGT4P, GAS2, CDC37P1, BTF3L4P2, RAB25, RNU6-322P, NPIPB8, ANGPTL1, PCDHGA2, NFKBIL1, MTUS2-AS1, SCARNA8, AC004134.1, AC010913.1, AL589745.1, DLG1-AS1, AP003392.5, AL121917.1, CACNA1B, HMGCS2, CDKL4, EVI2A, MARCHF4, AC012568.1, ZNF460-AS1, AC100827.5, FAT2, AC012181.1, FAM153CP, LINC00570, RPLP1P6, DUX4L50, AC104046.1, AJ003147.1, TCTEX1D4, RPL7P24, MIR6835, KCNJ2, SGO1-AS1, PKIA, AL451085.1, SUPT16HP1, SSTR2, KISS1R, AL137847.1, SUSD5, AL137800.1, VWF, AL157937.1, SLC25A21-AS1, PLA2G4D, AC003657.1, AL589993.1, AC012435.2, CD27, ADTRP, AL121820.1, SPDYE19P, GTF2IP7, NUS1P1, AC011317.1, DLX2-DT, AC102953.1, ADAMTSL1, H4C3, AC012157.2, AP006621.1, CT45A3, SARDH, LEF1-AS1, CFAP77, AL356356.2, HAPLN2, AL359541.2, FAM222A-AS1, CCDC181, AC104938.1, AC022417.1, SNORA22, C4BPAP2, AL358781.2, AP001189.4, AC023043.4, AJ009632.2, AP001767.3, LINC01524, AC004147.2, AC004771.2, AC005077.2, CTNNA1P1, WSCD1, CRYGN, FBXW10, AL512625.3, AC010973.1, TEX14, A3GALT2, AC106028.2, SULT2B1, AC032011.1, SEMA3B-AS1, AC018638.7, Z84485.1, | EED, CCDC85B, VAV1, ATRX, ATRX, EED, EED, HDAC1, HDAC2, EED, EED, ATP1A1, NINL, PSMB6, WDR61, RPN2, WSB2, GTF3C1, KLHDC2, PIN4, POLA2, SUZ12, EED, SUZ12, SNAI1, SUZ12, EED, JARID2, MTF2, JARID2, MTF2, SUZ12, RBBP4, EZH1, EZH2, SUZ12, EED, EED, SUZ12, CTNNB1, HIST1H3A, DNMT1, DNMT3A, DNMT3B, DNMT1, DNMT3A, DNMT3B, EED, AKT1, HIST1H3A, HIST1H3A, KDM5A, SUZ12, SIRT1, EPC1, E2F6, HDAC1, MYOD1, SUZ12, EED, RBL2, PHB2, PPP1R8, PPP1R8, SUZ12, WT1, DNMT1, RUNX3, PPARG, POU5F1, CDK1, SUZ12, EED, SUZ12, EED, RBBP4, SUZ12, EED, RBBP4, HOTAIR, HIST3H3, GATA4, EED, SUZ12, HIST3H3, RELA, RELB, SUZ12, RELA, RELB, EHMT1, SMYD3, EPC2, UHRF1, SNAI1, HDAC1, HDAC2, HDAC3, EED, YY1, EED, EED, RORA, RARA, SUZ12, TLE1, HDAC1, ASXL1, ESR1, CTNNB1, CTNNB1, MED1, SUV39H1, PJA1, KDM1A, PRMT5, SUV39H1, BTRC, SUZ12, SUZ12, EED, BRCA1, BRCA1, HOTAIR, EED, SUZ12, HOTAIR, CEP63, JARID2, SUZ12, EED, VCP, SUZ12, EED, NPLOC4, CPSF6, KIF5B, RAP1GAP, TDRD1, ACTG1, NUDT21, PLEC, UFD1L, THRAP3, CIT, WWP2, AEBP2, DHX9, ILF3, UBB, DDX5, SON, SNRPA, HIST4H4, UPF1, LIMA1, TAF15, FBXL18, RBBP4, PABPC4, U2AF1, SNRNP70, CALML3, POTEE, SNRPD3, YBX1, SNRPN, TUBB4B, ANXA4, LARP1, NCL, ARG1, SRSF1, C10orf12, TUBA1B, MTF2, LUC7L2, TRIM28, AR, IMMT, FUS, SRSF3, WWP1, RBM14, SNRPD2, U2AF2, ATP5B, ELAVL1, FLG, HIST1H2AE, HIST1H2AB, DDX3X, FABP5, TRA2A, EWSR1, SAFB2, FAM120A, PHF1, RALY, SRSF7, SNRPD1, SF3B1, HSPB1, SNRNP200, ZC3HAV1, COPB1, ILF2, KLC2, LRRC40, SRSF9, SERPINB12, CPSF1, GNB2L1, HSPA1A, HSPA1B, RBBP7, FIP1L1, PDCD6IP, SNRPF, SF3B2, ZNF638, ADAR, DBN1, PKM, SRSF10, EFTUD2, ACTN1, PHGDH, DSC3, ATP2A2, ASPSCR1, ATP5C1, EPRS, CHCHD3, IGLL5, KLC4, NUMA1, RBM39, SF3B3, TPI1, CNBP, DAZAP1, ERH, KLHL12, SLC25A10, SYNE1, PHF19, DHX36, FBL, SDCBP, AKAP8, CPSF7, LGALS7, MYLPF, PHF5A, DHX30, PDS5A, ANKFY1, CPSF2, DYNLT3, TRIM25, HNRNPAB, KHDRBS1, KLC1, LRPPRC, JARID2, MAGOH, NOP2, FAM208A, H2AFY, YBX3, KAT7, HYOU1, P4HB, SUZ12, EED, HIST3H3, CHD4, HDAC1, HDAC2, SUZ12, NCOA1, CDKN2C, FAT1, JARID2, MKI67, 44441, PHF1, RBBP4, RBBP7, SNRPB2, SSR1, API5, NOP14, EED, RBX1, HUWE1, EHMT2, OIP5, SUZ12, MKRN2, RPL36, C10orf12, ITSN2, MRPL2, CCDC93, FNBP1L, DNAAF5, CEP192, CDCA7L, WDR76, REEP4, AEBP2, DNAJC21, CENPV, C17orf96, TRIM37, SUZ12, PRDM14, DDB2, SUZ12, KAT2B, KAT2B, SIRT1, MDM2, ZBTB16, HNF4A, DANCR, CDK1, SUZ12, EED, DDX5, FBXW7, FBXW7, FOXM1, SUZ12, STUB1, AEBP2, C10orf12, C17orf96, EED, H3F3A, H3F3B, JARID2, MTF2, PHF1, PHF19, RBBP4, RBBP7, SIN3A, SUZ12, PBRM1, SUZ12, PHF1, AEBP2, DNMT1, EED, TRIM28, CHD4, ARID1A, SMARCA4, SMARCC2, SMARCA5, EP300, GTF2I, TRIM28, SUZ12, RBBP4, TRIM28, TRIM28, ARID1A, SMARCA2, SMARCA4, SMARCC2, SMARCC1, CHD4, MYCN, ZRANB1, SUZ12, EED, USP22, USP39, USP44, USP49, USP53, SUZ12, GTF3C1, FXR1, BIRC6, AHNAK, EED, NUMA1, ACIN1, SUPT6H, SUPT5H, SMARCA4, SMARCC1, SLTM, PBRM1, GTF3C4, RBBP4, JARID2, GTF3C2, TP53BP1, AEBP2, GTF3C3, KMT2D, ARID1A, SUPT16H, GTF3C5, DDB1, KPNA2, TRA2B, SSRP1, NAP1L1, TOP2A, PELP1, SMARCD1, ZNF281, RAD50, PAXBP1, SNW1, PLRG1, RBBP6, MRE11A, CHD7, BUB3, NOP58, PSIP1, AQR, CCAR2, SAP18, ZC3H11A, CCAR1, TRIM28, CRNKL1, PHF1, NOP56, MTA1, BPTF, ZCCHC3, RSF1, SIN3A, ZC3H18, SALL4, SMARCB1, IWS1, RBBP7, NUDT21, SMARCC2, KDM6A, BOD1L1, CPSF6, CTNNBL1, DPF2, CDK11B, UBTF, RANBP2, ZGPAT, CMAS, TRRAP, DEK, LIN28A, SMARCA1, PHF5A, WBP11, NUP93, MBD3, TOE1, PPP1R10, INTS1, BRD7, YTHDC1, YBX1, HDGFRP2, ZNF207, EZH1, EHMT2, KPNB1, CPSF4, PES1, FHL2, CFAP20, C17orf85, PHF14, EHMT1, AHCTF1, HMG20A, ZC3H14, TRMT1L, L1TD1, FHL3, C10orf12, HCFC1, CSNK2B, CHAF1A, RCC1, HELLS, NUP107, CSE1L, NOLC1, WDR61, NOL6, EIF3F, PHF19, WIBG, MCM7, SENP3, ZNF593, ATRX, ZRANB2, CTNNB1, KRR1, SENP2, TTC28, TCEB2, ZCCHC8, NOC2L, NHP2L1, MLF2, CHAF1B, HMGA1, ANKRD28, ZNF592, NUP160, FRG1, KPNA4, PRRC2C, RB1CC1, PDS5A, EP400, LIG3, LRWD1, ZNF524, CECR2, CWC15, TERF2, YWHAE, KMT2C, BRD2, ZNF157, MCM5, IPO5, PQBP1, SAP30BP, TRIM75P, FMR1, NUP85, TRIM4, CXorf56, PHIP, AAAS, EXOSC6, EIF3G, NUP205, ORC2, MTA3, RAD21, WDR36, SPEN, YWHAQ, LYAR, C19orf43, BRD4, REST, PPP1CC, ARMCX1, HMGA2, CACTIN, DMAP1, RBPJ, PLCD4, NUP35, CCNK, EXOSC8, NUP50, MCM2, C17orf96, ASCC3, CEBPZ, PDS5B, KAT7, GATAD2B, CENPJ, NCBP2, RFC1, POLDIP2, NUP98, NIFK, HIST2H2BC, LSM14B, EXOSC3, KIF20A, DPY19L1, SEC61A1, L3MBTL3, CHD2, HUWE1, KCTD6, LYRM1, PPP2R1A, EIF3K, GTF3C6, MTFR2, RPS19BP1, TCEB1, NOP16, MAGED1, TAF7, BRMS1, CELF1, BCL7C, ABHD16B, RGAG1, PTPN12, BAZ1A, LIG1, ZCCHC10, PCNP, CENPV, NPM3, CCP110, NIPSNAP1, TAF3, HIST1H1D, NAP1L4, MUC4, CHD1, TRIP12, SETD1A, BEND3, NACC1, TCEA1, ANGEL2, EIF2A, ABT1, ZFPM2, EXOSC9, FAM208A, MAGOHB, CDK13, MAZ, LRRC47, WDR43, C9orf114, USP42, PARD3B, PTCD3, PTPLAD1, TRIM21, C16orf71, RC3H2, LMAN2L, ZNF831, SETX, USP7, CHD8, SAP30, PCMTD2, KPNA3, ZEB2, PRMT5, HYDIN, LARP4B, RFC2, CLASRP, KDM5B, CCDC9, RYBP, ZMAT2, UHRF1, BRMS1L, YKT6, FANCI, PCNA, IGHV4OR15-8, KMT2A, CHD6, IBTK, TFAP2C, INTS2, NUP214, MECP2, GLTSCR2, CCNB1, ZMYM4, HAUS4, BCL9L, UBAP2L, GSG2, ARMCX3, UPF3B, CBLL1, VDR, CSNK1D, CCDC12, TPX2, FAM120A, SUN2, CUL4B, IQCD, PML, EYA1, RPGRIP1L, HDAC5, AIM1L, LRRC8E, BRD3, STAM2, MORF4L1, OLFML3, SNIP1, ARMCX2, RCOR3, KNOP1, ZNF462, ZC3H4, POLA2, AFG3L2, NCOA3, CHD3, CEP290, ZBTB48, TNRC6A, CRABP2, EIF3H, HAUS8, ING1, AIM1, TTC5, NFKBIZ, ZNF146, SPP1, UNC79, WNT8A, LEKR1, TOP2B, MTF2, SMC1A, XRN2, ASH2L, MYL6, HELB, GATAD2A, WDR18, SMARCD2, CAND1, MYBBP1A, CNOT1, CBX5, CEP44, AMZ2, SMCHD1, FSCN1, TAF2, HTATSF1, GCN1L1, INTS3, EXOSC4, TRIM71, ARID3B, NUP210, POF1B, POLE, RCOR1, ING4, TIAL1, ZNF148, LRRC59, RFC3, PABPC4, CCT6A, LRRC15, TRIM33, DAZAP1, TNKS1BP1, TERF2IP, CCDC141, NUDC, INTS4, CNTROB, GPATCH11, ZMAT3, SSH1, CTBP1, ARF4, MAD2L2, TSPYL2, MORF4L2, NOM1, MOV10, CDT1, ARMC8, OBSL1, C9orf43, IPO7, WBP4, STRAP, TMEM159, ANKS1A, LUZP1, CRISPLD1, DNA2, IKBIP, PRDM12, ALYREF, SMAD3, SUZ12, JARID2, SALL4, ZNF281, PAXBP1, RAB1A, TSC1, MAN1B1, MAN1A1, MAN2A1, SLC39A9, HDAC8, IGF2R, SEC23IP, POLE3, VPS51, TRAPPC5, PTEN, VPS45, TRAPPC2, RABGEF1, IGF1R, NCOR1, SEC24C, UBE2M, SEC31A, RICTOR, TBL1XR1, PHF1, MUC1, E2F1, HDAC1, MYC, RB1, ARMC12, ARMC12, FOXP4-AS1, SMURF2, USP44, USP44, NEAT1, SUZ12, SMAD2, CTNNB1, DNMT1, KDM1A, HDAC1, SMAD4, USP1, ZBTB16, AR, KRTAP10-9, MELK, USP36, USP7, EED, SUZ12, USP7, USP7, SMURF2, FBP1, SUZ12, EED, CXorf67, SUZ12, JARID2, AEBP2, | ARL6IP6, ASPM, ATAD2, AURKA, BUB1, C14orf106, C15orf42, C6orf173, CAPZA1, CBX7, CCDC92, CCNE2, CDC20, CDC7, CENPA, CENPF, CENPL, CENPN, CEP152, CHEK1, CKS2, COX7A1, CTTN, DBF4, DDX39, DEPDC1B, DNA2, DNMT1, DOT1L, DTL, DUSP3, E2F7, ECHDC2, ECT2, EED, ESPL1, FAM54A, FANCI, FBXO11, FBXO5, FBXW4, FEN1, GABPB2, GGH, GNG13, GNL2, GPR89A, GPX3, H2AFZ, HELLS, HMGB2, HMMR, HSPB2, KIAA0101, KIAA0494, KIAA1524, KIF11, KIF14, KIF2A, KIF4A, KNTC1, KPNA2, LMNB1, MAGOH, MASTL, MICB, MLF1IP, MSH6, MTHFD2, NDE1, NDRG2, NEK2, NUF2, NUFIP2, NUSAP1, NXT2, ORC6L, PINK1, PMAIP1, PPAT, PRC1, RACGAP1, RAD51AP1, RCC2, RFC4, RRM2, SFRS2, SGOL2, SMC1A, SMC4, SPRYD3, SRPK1, STIL, TENC1, TIMELESS, TMEM194, TMPO, TNS1, TNXB, TOM1L2, TOP2A, TOPBP1, TXNDC1, UBA6, UHRF1, USP1, VPS13D, WDHD1, WRN, ZBTB4, ZNF207, ZNF326, ZNF367, ZWILCH, ACVR2B, BIRC5, ATP5PF, ADGRB2, LDLRAD4, CAST, CDC20, CIDEA, CRH, CFD, DNM2, FOXD2, GABPA, GATM, HSF1, DNAJB1, FOXK2, IREB2, STMN1, LNPEP, TRIM37, NAB2, NF1, NPC1, OPRL1, CHMP1A, PDE7A, RET, RPA2, SRSF7, SLC11A1, HLTF, SOAT1, SREBF1, TCF12, NR1H2, USF1, YES1, SLC30A1, SEMA7A, CDK10, ALDH4A1, TMEM11, STBD1, MSC, COPB2, B4GALT6, TECR, POLR1C, KMT2B, RNF40, ZBTB40, PAN2, CTDSP2, PPIE, NCOA2, ARID3B, RAI1, UQCR11, MAPRE2, HNRNPUL1, SEPHS1, TPX2, DIP2C, PALLD, DOP1A, BOP1, NEDD4L, ZNF629, SSBP3, ARIH1, YIPF3, RTL8A, B3GAT3, ZBTB32, AHDC1, PRICKLE4, KCNIP2, CHST11, HSD17B11, HERC5, CCDC174, SHISA5, NCKIPSD, ASB1, DUOX1, CSNK1G1, TRMT13, MTRF1L, DNAJB12, MED18, ZNF280D, RASIP1, TMEM63B, KIF16B, ANKH, CELF4, ISY1, ZSWIM6, CTDSP1, PPCDC, TRAPPC11, BCAN, PIEZO2, PLEKHG2, MRPL14, CYBC1, GEMIN6, SNX22, PANK2, FHOD3, C18orf21, TMEM222, POMK, SPIRE2, UBE3D, FAM110B, KNSTRN, TMEM263, LENG9, FDX2, NIPA1, SPATA33, RAVER1, FAM241A, LOC145783, SASS6, LKAAEAR1, SLC39A11, RWDD4, DIPK2A, MARCHF8, BRAT1, YTHDF3, TMCO4, CATSPERE, ARHGAP30, LINC00323, LINC02843, TEX9, LINC01144, ACTG1P20, ANKRD33B, MIR638, MIR639, MSC-AS1, TOMM6, LOC100289230, LINC01578, ISY1-RAB43, MIR4733, FAM47E-STBD1, HLTF-AS1, YTHDF3-AS1, ADAMTS1, ADRB2, ALDH1A1, APC, AXL, BRCA1, CCND1, CCNE1, CDH1, CDKN1A, CDKN2A, CDKN2B, CIITA, DACT3, FBXO32, FOXC1, HDAC1, HOXB13, MMP2, PCNA, PEBP1, PIP5K1C, RPLP0, RUNX3, SATB1, SFRP1, SNAI2, SSTR1, TP53, TWIST1, VDR, | RORA, PHGDH, PTPN12 | GNG13, MTHFD2, FHOD3, CDKN1A, CDKN2B, FBXO32, TWIST1 | RORA, PHGDH, PTPN12, GNG13, MTHFD2, FHOD3, CDKN1A, CDKN2B, FBXO32, TWIST1 |

**Supplementary Table S7. Clinical relevance of the circGSK3B/EZH2/RORA axis in GC.**

| **Clinicopathologic**  **parameter** | **Number** | **Number of patients(circRNA)** | | **p value** | **Number of patients (RORA)** | | **p value** | **Number of patients (EZH2)** | | **p value** |
| --- | --- | --- | --- | --- | --- | --- | --- | --- | --- | --- |
|  |  | **Low**  **n=28** | **High**  **n=28** |  | **Low**  **n=28** | **High**  **n=28** |  | **Low**  **n=28** | **High**  **n=28** |  |
| **Age** |  |  |  |  |  |  |  |  |  |  |
| <60y | 20 | 11 | 9 | 0.577 | 8 | 12 | 0.265 | 13 | 7 | 0.094 |
| ≥60y | 36 | 17 | 19 |  | 20 | 16 |  | 15 | 21 |  |
| **Gender** |  |  |  |  |  |  |  |  |  |  |
| Male | 41 | 21 | 20 | 0.763 | 22 | 19 | 0.365 | 18 | 23 | 0.131 |
| Female | 15 | 7 | 8 |  | 6 | 9 |  | 10 | 5 |  |
| **Tumor size** |  |  |  |  |  |  |  |  |  |  |
| <3cm | 16 | 4 | 12 | 0.018* | 5 | 11 | 0.076 | 12 | 4 | 0.018* |
| ≥3cm | 40 | 24 | 16 |  | 23 | 17 |  | 16 | 24 |  |
| **Tumor site** |  |  |  |  |  |  |  |  |  |  |
| Proximal | 25 | 12 | 13 | 0.788 | 11 | 14 | 0.420 | 10 | 15 | 0.179 |
| Non-proximal | 31 | 16 | 15 |  | 17 | 14 |  | 18 | 13 |  |
| **Lymph node metastasis** |  |  |  |  |  |  |  |  |  |  |
| N0 | 22 | 7 | 15 | 0.029* | 8 | 14 | 0.101 | 16 | 6 | 0.006* |
| N1-N3 | 34 | 21 | 13 |  | 20 | 14 |  | 12 | 22 |  |
| **TNM stage** |  |  |  |  |  |  |  |  |  |  |
| I-II | 24 | 8 | 16 | 0.031* | 8 | 16 | 0.031* | 17 | 7 | 0.007* |
| III | 32 | 20 | 12 |  | 20 | 12 |  | 11 | 21 |  |
| **Blood vessel invasion** |  |  |  |  |  |  |  |  |  |  |
| Negative | 39 | 17 | 22 | 0.146 | 18 | 21 | 0.383 | 23 | 16 | 0.042* |
| Positive | 17 | 11 | 6 |  | 10 | 7 |  | 5 | 12 |  |

^*^P<0.05 indicates a significant relationship among the variables.
