# Supplementary material for: CircGSK3B promotes RORA expression and suppresses gastric cancer progression through the prevention of EZH2 trans-inhibition
Source: J Exp Clin Cancer Res. 2021 Oct 19;40:330. doi: 10.1186/s13046-021-02136-w (PMC8524915; doi:10.1186/s13046-021-02136-w)
Supplement: Supplementary file 3 — Additional file 3: Figure S1. Expression profiles of circRNAs. (a) RT-PCR assay with divergent primers showing the detectable (left panel) and undetectable (right panel) circRNAs in cultured MKN45 cells. (b, c) The genomic locus of hsa_circ_0036287 (b) and hsa_circ_0000423 (c). The expression level of hsa_circ_0036287 or hsa_circ_0000423 was assessed via RT-PCR assay and Sanger sequencing. Arrows represent divergent primers targeting its genome region. Figure S2. Lentivirus-mediated knock down selection and the mass spectrometric results pulled down by the circGSK3B. (a) Knockdown efficiency of three sequences targeting circGSK3B. sh-circGSK3B #2 has the highest knockdown efficiency and is used for subsequent experiments. (b) Knockdown efficiency of two sequences targeting RORA. sh-RORA #1 has the highest knockdown efficiency and is used for subsequent experiments. (c) MS assay showing the EED peptides pulled down by circGSK3B. (d) MS assay showing the SUZ12 peptides pulled down by circGSK3B. Figure S3. circGSK3B targeted RORA expression through EZH2-mediated epigenetic regulation in GC cells. (a) Binding transcription factors to RORA promoter region was assessed by UCSC. (b) The effect of circGSK3B overexpression and GSK126 (EZH2 inhibitor) on RORA, H3K27me3, β-catenin, P-β-catenin, and CCND1 (Canonical target gene of β-catenin) assessed by western blot assay in MKN45 and AGS cell lines. (c, d) Relative enrichment of EZH2 and its catalytic histone marks H3K27me3 on the promoter region of RORA gene was evaluated by ChIP-qPCR assays in MKN45 and AGS cells. Primer 1 (− 61 ∼ + 45), Primer 2 (− 530 ∼ − 380), Primer3 (− 1019 ∼ − 870), Primer 4 (− 1562 ∼ − 1395) and Primer 5 (− 1973 ∼ − 1731). IgG was used as a negative control. Figure S4. Representative images (upper panel) and quantification (lower panel) of immunohistochemical staining showing the expression of Ki-67 and CD31 within xenograft tumors formed by hypodermic injection of MKN45 cells stably transfected with [file 13046_2021_2136_MOESM3_ESM.docx]

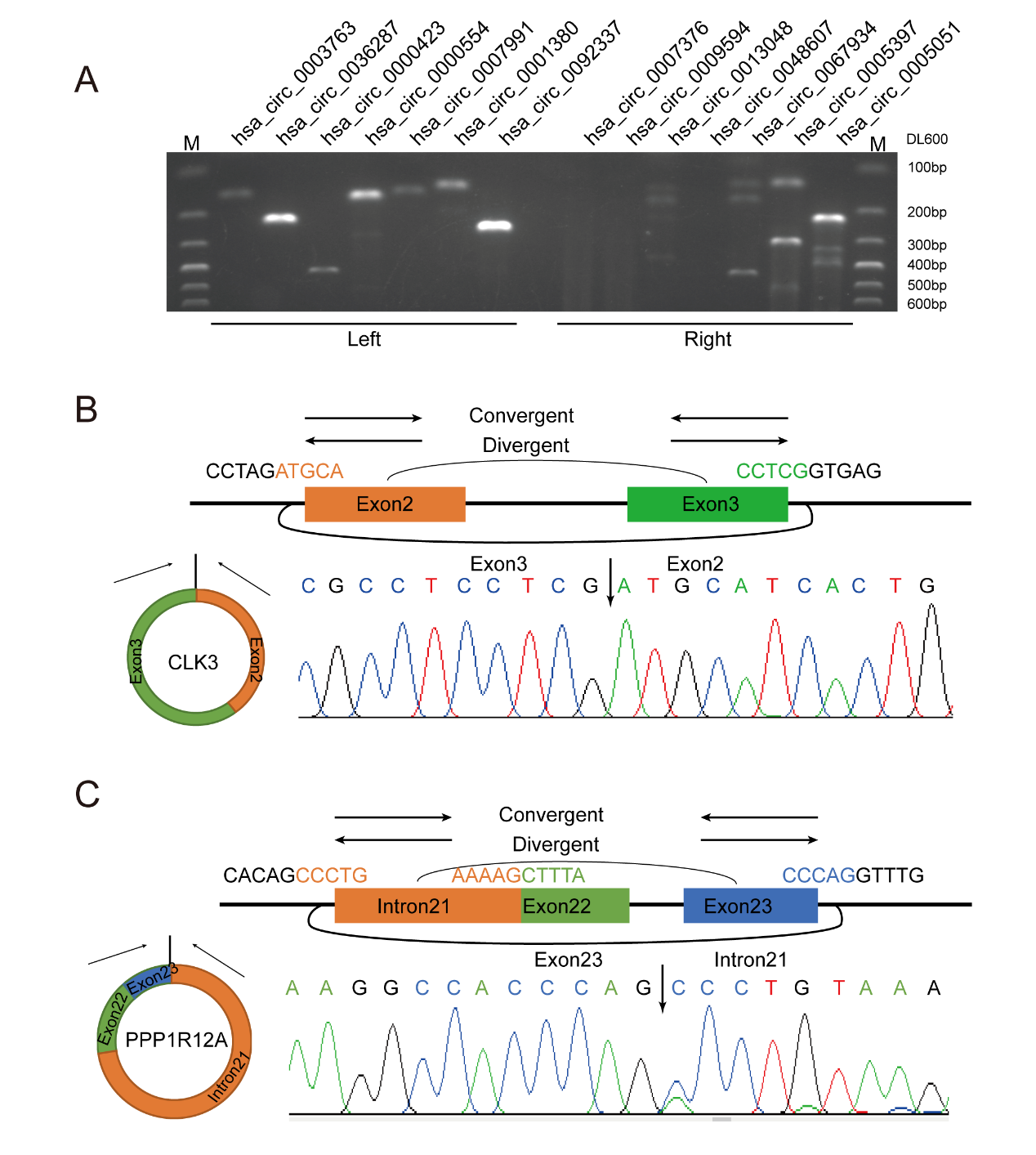


**Figure S1. Expression profiles of circRNAs. (a**) RT-PCR assay with divergent primers showing the detectable (left panel) and undetectable (right panel) circRNAs in cultured MKN45 cells. (**b, c**) The genomic locus of hsa_circ_0036287 (b) and hsa_circ_0000423 (c). The expression level of hsa_circ_0036287 or hsa_circ_0000423 was assessed via RT-PCR assay and Sanger sequencing. Arrows represent divergent primers targeting its genome region.


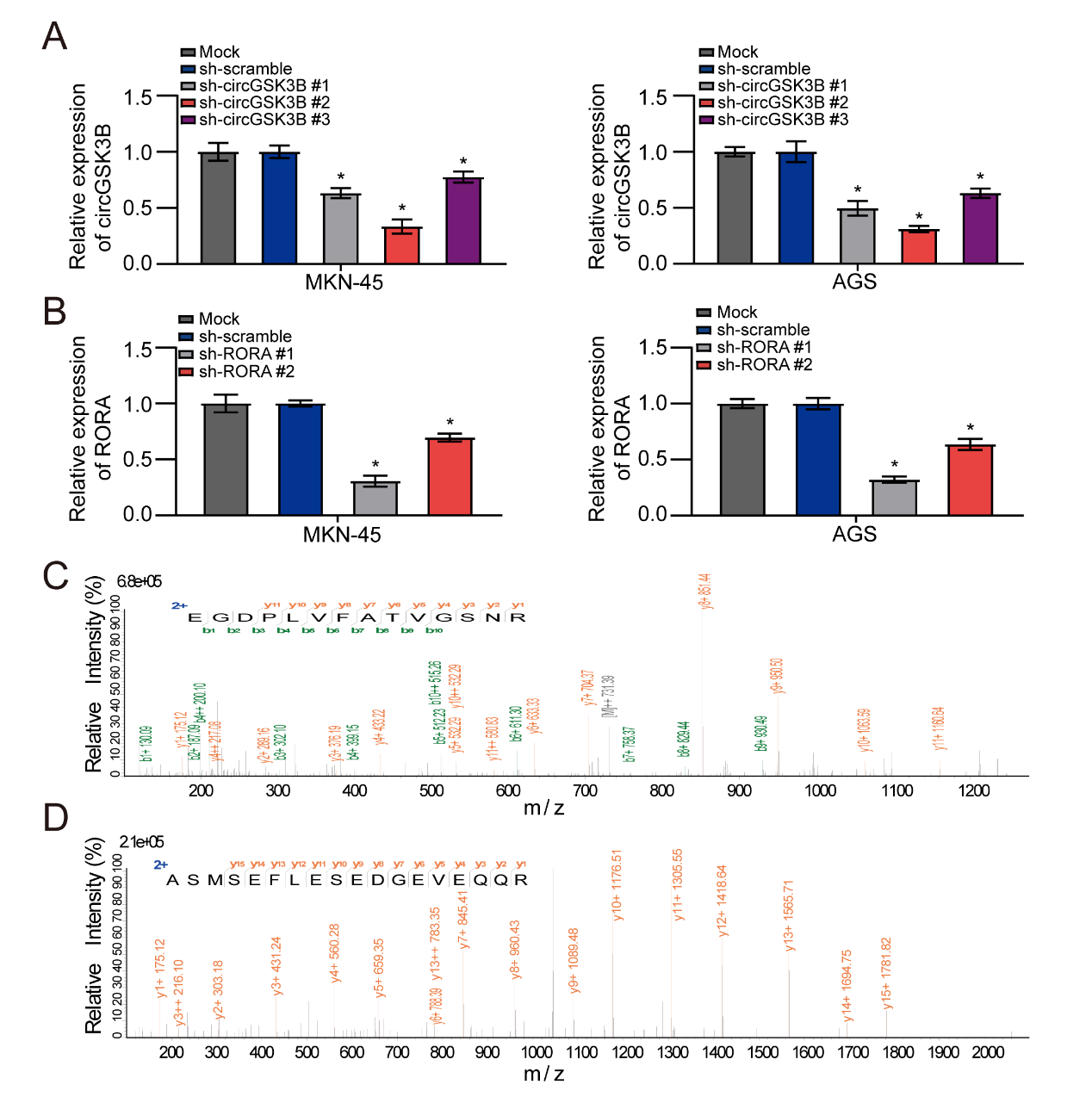


**Figure S2. Lentivirus-mediated knock down selection and the mass spectrometric results pulled down by the circGSK3B. (a)** Knockdown efficiency of three sequences targeting circGSK3B. sh-circGSK3B #2 has the highest knockdown efficiency and is used for subsequent experiments. **(b)** Knockdown efficiency of two sequences targeting RORA. sh-RORA #1 has the highest knockdown efficiency and is used for subsequent experiments. **(c)** MS assay showing the EED peptides pulled down by circGSK3B. **(d)** MS assay showing the SUZ12 peptides pulled down by circGSK3B.


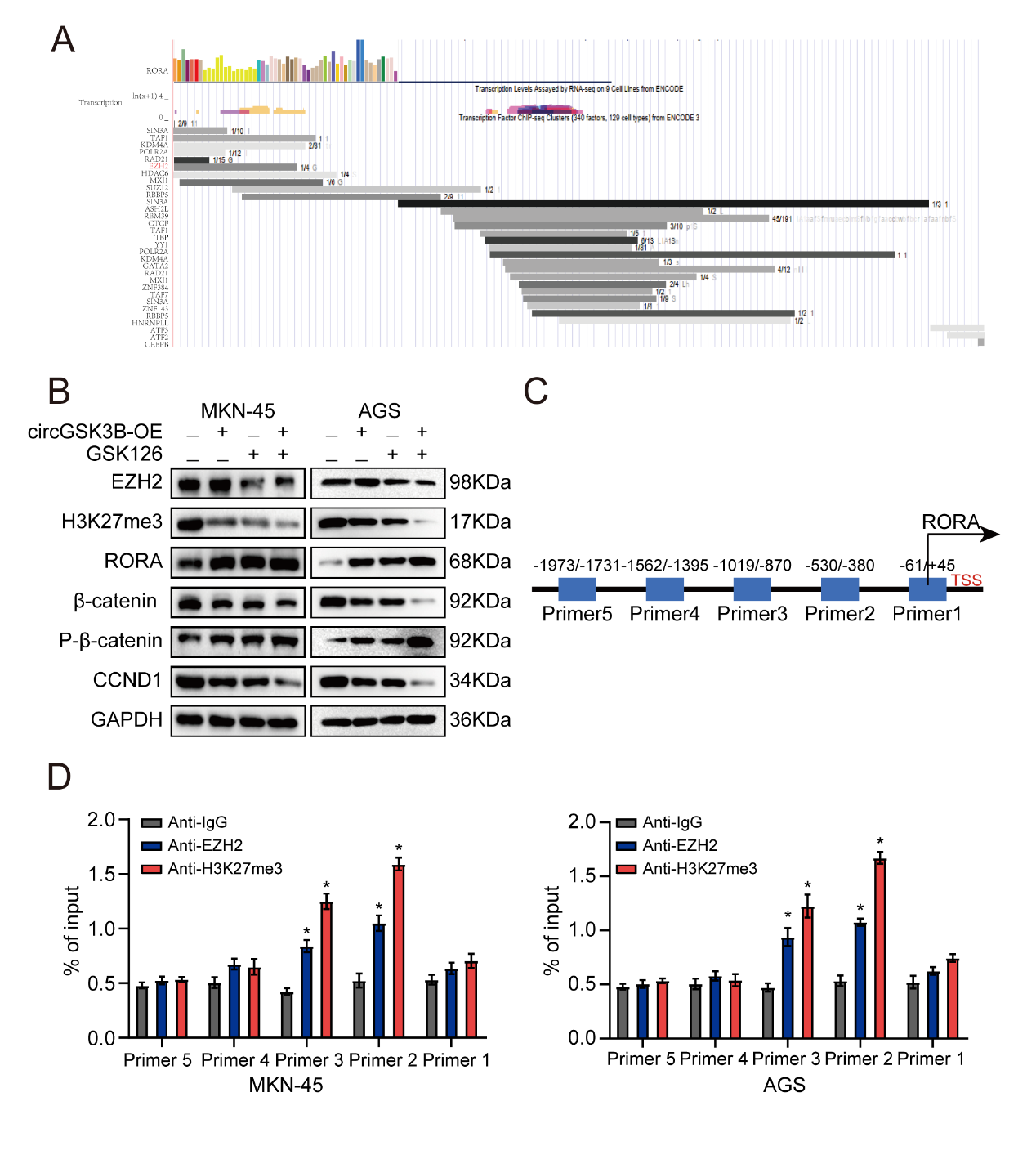


**Figure S3. circGSK3B targeted RORA expression through EZH2-mediated epigenetic regulation in GC cells. (a)** Binding transcription factors to RORA promoter region was assessed by UCSC. (**b**) The effect of circGSK3B overexpression and GSK126 (EZH2 inhibitor) on RORA, H3K27me3, β-catenin, P-β-catenin, and CCND1 (Canonical target gene of β-catenin) assessed by western blot assay in MKN45 and AGS cell lines. (**c, d**) Relative enrichment of EZH2 and its catalytic histone marks H3K27me3 on the promoter region of RORA gene was evaluated by ChIP-qPCR assays in MKN45 and AGS cells. Primer 1 (-61 ∼ +45), Primer 2 (-530 ∼ -380), Primer3 (-1019 ∼ -870), Primer 4 (-1562 ∼ -1395) and Primer 5 (-1973 ∼ -1731). IgG was used as a negative control.


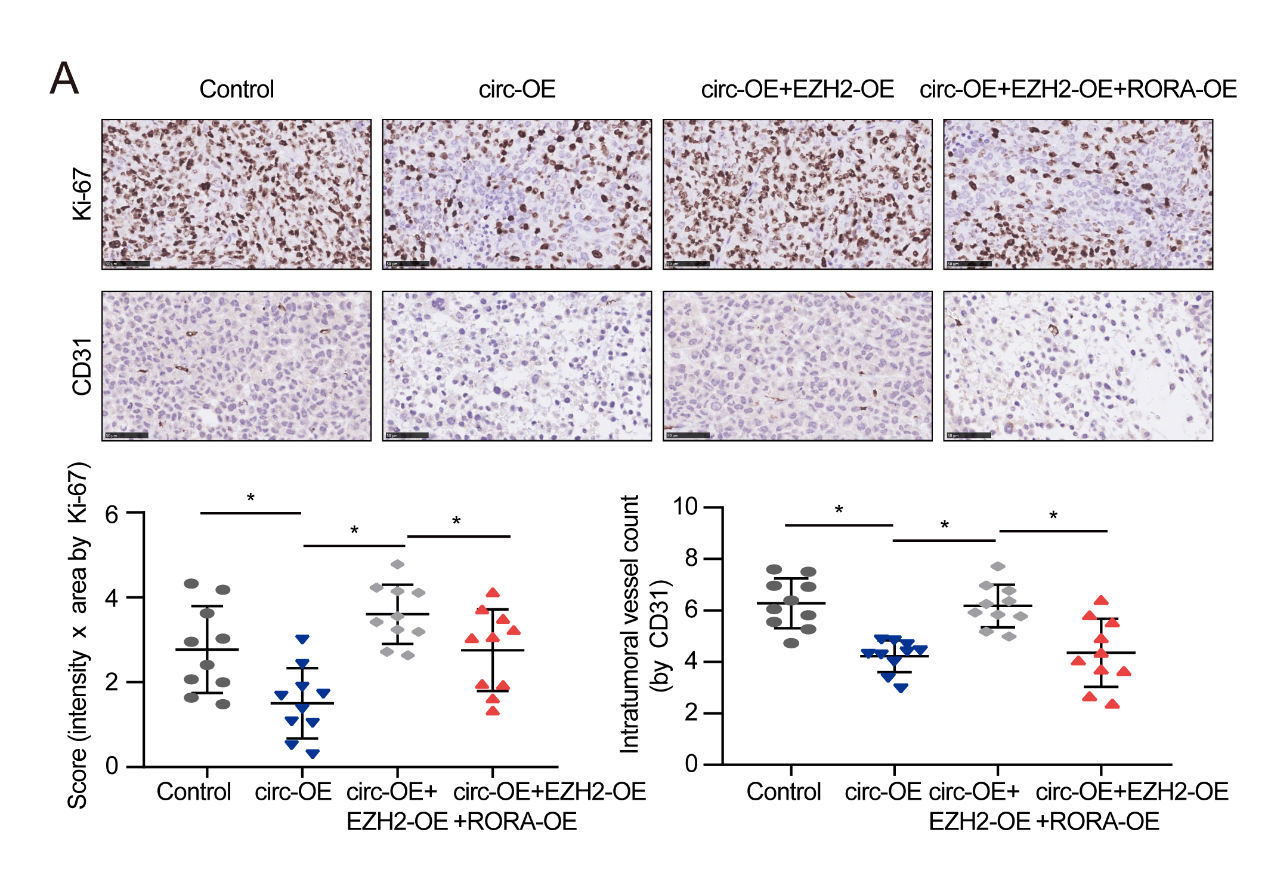


**Figure S4.** Representative images (upper panel) and quantification (lower panel) of immunohistochemical staining showing the expression of Ki-67 and CD31 within xenograft tumors formed by hypodermic injection of MKN45 cells stably transfected with circ-Mock, circGSK3B-OE, circGSK3B-OE+EZH2-OE, circGSK3B-OE + EZH2-OE + RORA+OE.
